# Neurotoxic Effects of Linalool and β-Pinene on *Tribolium castaneum* Herbst

**DOI:** 10.3390/molecules22122052

**Published:** 2017-11-24

**Authors:** Nerlis Pajaro-Castro, Karina Caballero-Gallardo, Jesus Olivero-Verbel

**Affiliations:** 1 Environmental and Computational Chemistry Group, Zaragocilla Campus, School of Pharmaceutical Sciences, University of Cartagena, Cartagena 130016, Bolivar, Colombia; npajaroc@unicartagena.edu.co (N.P.-C.); kcaballerog@unicartagena.edu.co (K.C.-G.); 2 Medical and Pharmaceutical Sciences Group, School of Health Sciences, Department of Medicine, University of Sucre, Sincelejo 700003, Sucre, Colombia

**Keywords:** gene expression, insects, monoterpenes, repellents

## Abstract

Effective, ethical pest control requires the use of chemicals that are highly specific, safe, and ecofriendly. Linalool and β-pinene occur naturally as major constituents of the essential oils of many plant species distributed throughout the world, and thus meet these requirements. These monoterpenes were tested as repellents against *Tribolium castaneum*, using the area preference method, after four hours of exposure and the effect transcriptional of genes associated with neurotransmission. Changes in gene expression of acetylcholinesterase (Ace1), GABA-gated anion channel splice variant 3a6a (Rdl), GABA-gated ion channel (Grd), glutamate-gated chloride channel (Glucl), and histamine-gated chloride channel 2 (Hiscl2) were assessed and the interaction with proteins important for the insect using in silico methods was also studied. For linalool and β-pinene, the repellent concentration 50 (RC_50_) values were 0.11 µL/cm^2^ and 0.03 µL/cm^2^, respectively. Both compounds induced overexpression of Hiscl2 gen in adult insects, and β-pinene also promoted the overexpression of Grd and the Ace1 gene. However, β-pinene and linalool had little potential to dock on computer-generated models for GABA-gated ion channel LCCH3, nicotinic acetylcholine receptor subunits alpha1 and alpha2, and putative octopamine/tyramine receptor proteins from *T. castaneum* as their respective binding affinities were marginal, and therefore the repellent action probably involved mechanisms other than direct interaction with these targets. Results indicated that β-pinene was more potent than linalool in inducing insect repellency, and also had a greater capacity to generate changes in the expression of genes involved in neuronal transmission.

## 1. Introduction

Biopesticides are gaining increased attention and interest among those concerned with environmentally friendly, safe, and integrated crop management approaches [1,2] and, correspondingly, nature has provided us with a variety of chemotherapeutic agents [3]. Among them, essential oils (EOs) and their constituents affect biochemical processes, specifically disrupting the endocrinological balance of insects. In fact, some plants biosynthesize EOs to protect themselves from insects and may disrupt the process of morphogenesis through neurotoxicity, or by acting as growth regulators [1] and interfering with the basic metabolic and physiology of insects [4].

Terpenes are a class of plant secondary metabolites that have a distinct structure and function, and are considered to be important agents in the medicinal uses of aromatic plants [3]. Monoterpenes, a structurally diverse group of phytochemical compounds, are the major constituent of EOs. In recent years, interest in understanding the pharmacological actions of these molecules has increased. Studies have demonstrated that these naturally occurring molecules can modulate the functional properties of various types of voltage and ligand-gated ion channels, and thus can be used as alternatives to synthetic insecticides against pests on stored products [5].

**Figure 1:**
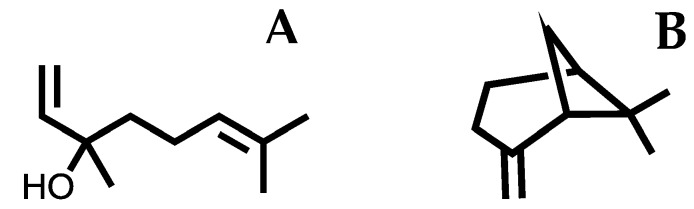
2D chemical structures of linalool (**A**) and β-pinene (**B**).

Linalool and β-pinene are two alcohol and hydrocarbon volatile monoterpenes [6] reported to be major components of EOs in various aromatic species [7] (Figure 1). Linalool is produced by plants and is used in cosmetic products, perfumes, and flavorings. Furthermore, it has been shown to have antimicrobial activity and insect-repellant properties that act on the central nervous system (CNS) [6]. Linalool is slightly volatile with a pleasant aroma associated with lavender and laurel fragrances [3,8]. The aim of this study was to evaluate the effects of linalool and β-pinene in the expression of genes related to neurotransmission on *T. castaneum*, and assesses the interaction with proteins important for the insect using in silico methods.

## 2. Results

### 2.1. Repellent Activity

**Figure 2:**
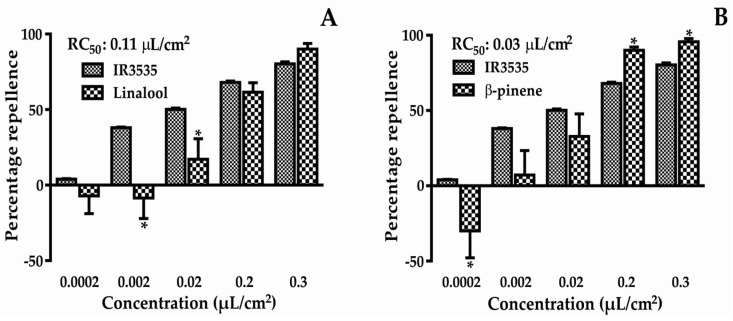
Percentage repellence of linalool (**A**) and β-pinene (**B**) against *T. castaneum*. * Statistically significant compared to control (*p* < 0.05). (**A**) t-value = 1.050; F-value = 4.411; (**B**) t-value = 2.646; F-value = 4.133.

The percentage repellency results from the chemicals tested are presented in Figure 2. At the lowest concentration, attractive activity was found for both compounds. However, at higher concentrations, these compounds showed strong repellent activity, with repellent concentration 50 (RC_50_) values of 0.11 and 0.03 µL/cm^2^ for linalool and β-pinene, respectively. The RC_50_ of the positive control was 0.02 µL/cm^2^. The two-way ANOVA results showed that there was interaction between the different concentrations evaluated, therefore, the repellent effect was dependent on the concentration.

### 2.2. Gene Expression

**Figure 3:**
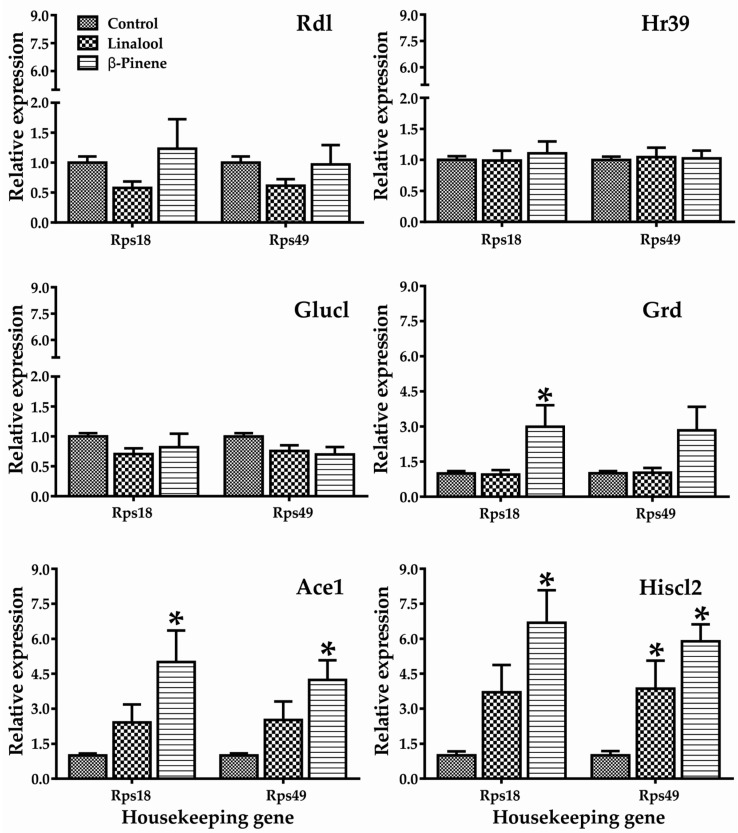
Relative mRNA expression of selected genes in adults of *T. castaneum* after 4 h exposure to different concentrations of linalool and β-pinene. Within each gene, relative expression was normalized against the control (Value = 1). * Significant effects compared to control. Rdl: t-value = 1.636; F-value = 0.542; Hr39: t-value = 1.718; F-value = 0.903; GluCl: t-value = 0.450; F-value = 0.579; Grd: t-value = 0.042; F-value = 0.065; Ace1: t-value = 2.559; F-value = 0.924; HisCl2: t-value = 3.157; F-value = 0.715.

In this study, changes in the gene expression of *T. castaneum* adults are presented in Figure 3. Both constituents behaved differently, and showed statistically significant differences in the expression of genes such as Grd, Ace1, and Hiscl2. Although insects exposed to linalool did not show significant differences in the expression of the gene Ace1, a tendency to increase was observed in both housekeeping genes. The two-way ANOVA showed that there was no interaction between the genes, therefore, the gene expression was independent for each gene.

### 2.3. Homology Modeling and Validation

The best proteins obtained in the homology modeling process were loaded in Protein Model Data Base (PMDB). The validation results obtained using the different servers demonstrated the quality of the models. ProQ rated the obtained models as fairly, very, and extremely good (Table 1).

The results of the validation process obtained by RAMPAGE showed that the studied proteins had a number of residues in favored and allowed regions of over 90% (Figures S1–S13). Figures S14–S26 depict the results obtained using QMean, which were a measure of the degree of nativity of a structure for a particular protein. These results showed values with a negative sign, indicating scores lower than those obtained for the average experimental structures.

### 2.4. Molecular Docking

A computational approach was carried out to identify possible targets for linalool and β-pinene, with a particular focus on the *T. castaneum* proteins involved in neurological responses. The results are presented in Table 2. β-pinene produced better interactions than linalool, with affinity values ranging from −7.7 to −5.0 Kcal/mol. However, these affinity values were low when compared to those obtained from known inhibitors.

The 3D structure of the proteins and amino acids involved in the protein-ligand interaction are shown in Figure S27. Linalool and β-pinene bind to the same site and therefore interact with the same type of amino acids in all the studied complexes, except those formed with the carboxylic ester hydrolase and the glutamate-gated chloride channel proteins where the chemicals were coupled on different sites of the protein. The major amino acids identified in the protein-ligand interactions were ILE, LEU, THR, SER, and VAL, with a frequency of occurrence greater than 40%. The main protein–ligand interactions identified were hydrophobic and hydrogen bonding.

## 3. Discussion

Natural insect repellent products have become a viable alternative to synthetic repellents [12,13] as they provide less risk to the environment and to human health [7]. In this paper, two main EO compounds were studied to evaluate repellent activity, gene expression, and molecular docking. β-pinene showed greater repellent activity than linalool (Figure 2), and both had smaller RC_50_ values than IR3535, although these were comparable with the positive control.

β-pinene was more potent against *T. castaneum* than linalool. This compound is an oxygenated monoterpene which is abundantly present in the environment, and is widely found in plants as a constituent of EOs [14]. It has been reported with acute toxicity to insects of *T. castaneum* after three days of exposure [15]. Meanwhile, linalool is a monoterpene of 10 carbon atoms and an alcohol group [16] that has been reported as a mosquito (*Aedes aegypti*) [17] and beetle repellent (*T. castaneum* and *Dominica Rhyzopertha*) [18], which coincides with the effects observed in this study.

Linalool and β-pinene are insect repellents; however, the mechanisms by which these compounds function are not fully understood. It is only known that monoterpenes can act on various insect and mammalian receptors, especially at the level of the nervous system, and specifically on gamma-aminobutyric acid (GABA)-gated chloride channels; octopamine, tyramine, and nicotinic acetylcholine (nAChR) receptors; acetylcholine esterase; sodium channels; and other targets. Several different monoterpenoids have been reported to bind to ionotropic GABA receptors in humans, rodents, and insects in previous research [1,5,19]. Allelochemical defense systems in insects allow the rapid elimination of ingested toxic substances, and/or the fat bodies, therefore facilitating the detoxification of contaminants penetrating the cuticular or tracheal structures through enzymes—such as P450, Glutathione-S-transferases, and esterases—which are typically concentrated in the insect midgut [1]. However, some of the most important targets of insecticidal agents are inhibitory neurotransmitter receptors such as invertebrate gamma-aminobutyric acid (GABA)-gated chloride channels (GABACls) and glutamate-gated chloride channels (GluCls), which are related to changes in behavior [20].

Repellent activity is related to changes in the behavior of insects, which are caused by effects on the CNS. The histamine-gated ion channel is a member of the cys-loop ligand-gated ion channel (cys-loop LGIC) superfamily [21], and has two subunits—HisCl1 and HisCl2—that control the phototransduction of invertebrates [22]. Outside of phototransduction, histamine also appears to be the neurotransmitter found in some mechanosensory neurons in *Drosophila* as well as in lobster stomatogastric, cardiac, and olfactory neurons [23,24]. Adult insects exposed to linalool and β-pinene overexpressed Hiscl2 gen (Figure 3), most likely suggesting that the function of this gene could be one of the mechanisms involved in the repellent action of these monoterpenes. However, these compounds have little in silico ability to bind to this protein, with affinity values of −5.0 ± 0.2 and −5.7 ± 0.0 Kcal/mol for linalool and β-pinene, respectively (Table 2), leaving room for the involvement of modulatory mechanisms not related to direct binding.

Monoterpenes are capable of quickly penetrating insect cells as they are volatile and lipophilic compounds that interfere with physiological functions so their modes of action or mechanisms of resistance are especially complex [1,5]. For instance, they interact with the functioning of GABA synapses [25], or change the activity of voltage gated and/or ligand-gated ion channels in the CNS, thus impairing the activity of neuronal enzymes such as Ace [26].

Compared to β-pinene, linalool exhibited less activity in terms of gene expression, despite some potential to interact with proteins involved in synapsis (Figure S27b). On the other hand, β-pinene induces overexpression of Grd and Ace1 in *T. castaneum* adults (Figure 3). The ionotropic receptors of *T. castaneum* function as GABA-gated chloride channels (GABACls-Grd), receptor members of the family of the Cys-loop ligand-gated ion channels, which mediate fast inhibitory synaptic transmission [22,27]. On the other hand, the canonical biological function of Ace is to terminate impulse transmission at cholinergic synapses by rapidly hydrolyzing the neurotransmitter acetylcholine in animals; thus, it is the target of anticholinesterase insecticides [28]. Although it is clear that the evaluated monoterpenoids had little capacity to directly interact in silico with several proteins related to neurotransmission (Table 2, Figure S27), the activation of gene expression indicated that other alternative mechanisms were indirectly modulating their repellent activities.

The use of molecular tools together with in silico approaches assists in gaining insight into toxicity mechanisms [29,30,31]. In this investigation, in addition to evaluating gene expression, the interactions of two monoterpenes with important proteins in neurotransmission were also studied. Homology modeling was employed to construct the three-dimensional structure of proteins prior to molecular docking. The quality of the models was evaluated with QMean, ProQ, and RAMPAGE (Table 1, Figures S1–S26), and LGscore and MaxSub values implied that the obtained models were good [32]. The QMean *Z*-score uses structures that are solved by X-ray crystallography as a reference to estimate the absolute quality of a model [33]. For the models in this study, the QMean *Z*-score was in the range of −10.99 to −4.70, showing that these models varied in quality; conversely, however, the results obtained with RAMPAGE and ProQ suggested that the models had the appropriate quality [34,35,36].

The application of computational approaches is often used for the study of the toxicological profiles of new candidate insecticidal agents [37]. The docking results revealed that linalool and β-pinene had little ability to interact with the *T. castaneum* proteins involved in the neurotransmission process (Table 2) as the binding affinity were between −5.0 and 6.0 Kcal/mol, values far from those obtained for known inhibitors, which are around −9.0 Kcal/mol (Table 2) [9,10,11]. Interestingly, protein–ligand interactions for β-pinene and linalool also shared some residues with Ivermectin [38]; suggesting a common binding site. Although the evaluated proteins were related to the action of several chemical compounds on insects, in this in silico study, linalool and β-pinene showed very low affinity values on the proteins, indicating that repellent action probably involved alternative mechanisms.

## 4. Materials and Methods

### 4.1. Insect Rearing

The wild-type strain of *Tribolium castaneum* Herbst was taken from a stock colony maintained in our laboratory. The identification of *T. castaneum* was performed according to the morphological characteristics reported by Dönitz et al. [39]. Insects were reared on a diet of flakes and ground oats (70:30) at 26 ± 2 °C, with a 70% to 85% relative humidity and a 10:14 h light:dark photoperiod [2,6,7]. Then, two to four week old healthy adults were randomly chosen for bioassays.

### 4.2. Materials

Linalool (>99%) and β-pinene (>95%) were purchased from Sigma-Aldrich (Steinheim, Germany). Acetone reagent (≥99.5%) was also purchased from Sigma-Aldrich.

### 4.3. Repellent Activity

The experiments were carried out on adults of *T. castaneum* under laboratory conditions. The repellent activity of linalool and β-pinene was evaluated using the area preference method [20,40]. Test areas consisted of 9 cm Albet DP 597125 filter paper cut in half (31.8 cm^2^). As experimental containers, Petri dishes (9 cm in diameter) were used to house 20 *T. castaneum*. Linalool and β-pinene were serially dissolved in acetone (0.0002, 0.002, 0.02, 0.2 and 0.3 µL/cm^2^). Five hundred µL of the solution were uniformly applied to a half-filter paper disc to five different testing concentrations. The other half of the remaining filter paper was treated with 500 µL of acetone and used as the control vehicle. Then, the half discs were air-dried for about 10 min to allow solvent evaporation. During each test, 20 *T. castaneum* adults without regard to gender were released at the center of the disk, then covered quickly with a dish cover and placed in darkness. Counts of the insects present on each side of the filter paper were made after 4 h of exposure. As a positive control, a 15% formulation of IR3535 ethyl 3-(*N*-acetyl-*N*-butylamino)propionate [2] was used. The percent repellency (PR) of each compound/commercial repellent was then calculated using the formula Equation (1).

PR = [(Nc − Nt)/(Nc + Nt)] × 100
(1)

where Nc and Nt are the number of insects on the untreated (control) and treated areas, respectively. Three replicates were used for each tested concentration of the main compound, and each assay was repeated twice.

After the experiment was conducted, the median repellent concentration (RC_50_) for each compound was obtained by Probit analysis (RC_50_ = 0.11 µL/cm^2^ for linalool, and RC_50_ = 0.03 µL/cm^2^ for β-pinene) and was used for gene expression assays.

### 4.4. Gene Expression Assays

Adult control and exposed organisms were directly frozen with liquid nitrogen and preserved in RNAlater. Total RNA was isolated using an RNeasy Mini Kit (Qiagen, Valencia, CA, USA) according to the manufacturer’s protocols. RNA concentrations were measured using a NanoDrop 2000 spectrophotometer (Thermo Scientific, Wilmington, DE, USA) and the quality was verified by absorbance ratio 260/280 nm. cDNA was obtained through the QuantiTect^®^ Reverse Transcription Kit (Qiagen Inc., Valencia, CA, USA) according to the manufacturer’s protocols. A real-time polymerase chain reaction (RT-PCR) was performed on a StepOne Plus real-time PCR system (Applied Biosystems, Foster City, CA, USA) apparatus. Six genes were analyzed (Table 3) [41,42,43]. Gene expression was normalized to ribosomal protein 18 (Rps18) and ribosomal protein 49 (Rps49) [44]. Primer sequences are presented in Table 3. RT-PCR was performed with a Maxima SYBR Green/ROX qPCR Master Mix (Thermo Scientific, Waltham, MA, USA). The ΔΔCT was used to calculate relative gene expression. All experiments were run in duplicate [45,46,47].

### 4.5. Homology Modeling and Validation

The amino acid sequences of the proteins from *T. castaneum* were obtained from Uniprot [48] in FASTA format with the following access numbers E7DN61, E7DN62, A8DMU1, A8DMU2, A8DMU3, A8DMU5, A8DMU7, A8DMU8, D2A6H1, A8DIP3, A8DIQ7, A8DMU9, and D6WB14. Three-dimensional structures of carboxylic ester hydrolase, carboxylic ester hydrolase 2, gamma-aminobutyric acid (GABA)-RDL, GABA-GRD, GABA-gated ion channel LCCH3, glutamate-gated chloride channel, histamine-gated chloride channel 1, histamine-gated chloride channel 2, hormone receptor in 39-like, nicotinic acetylcholine receptor subunit alpha1, nicotinic acetylcholine receptor subunit alpha2, pH sensitive chloride channel, and putative octopamine/tyramine receptor proteins were constructed by homology modeling using (PS)2-v2: Protein Structure Prediction Server [49,50], Phyre2 [51], and I-Tasser [52]. The best model generated by each prediction server was selected to validate the model quality [33]. The quality of the models was evaluated using the RAMPAGE [53], ProQ (Protein Quality Predictor), and QMEAN [31,54]. The best structure of each protein obtained after the validation procedure was subjected to structural analysis using the molecular graphics system PyMOL [33] and saved in the Protein Model DataBase (PMDB) [32]. PM0081096, PM0081123, PM0081100, PM0081098, PM0081126, PM0081097, PM0081124, PM0081099, PM0081101, PM0081125, PM0081129, PM0081127, and PM0081128 were assigned as the identifiers for the structures carboxylic ester hydrolase, carboxylic ester hydrolase2, GABA-RDL, GABA-GRD, GABA-gated ion channel LCCH3, glutamate-gated chloride channel, histamine-gated chloride channel 1, histamine-gated chloride channel 2, hormone receptor in 39-like, nicotinic acetylcholine receptor subunit alpha1, nicotinic acetylcholine receptor subunit alpha2, pH sensitive chloride channel, and putative octopamine/tyramine receptor, respectively.

### 4.6. Molecular Docking

Prior to molecular docking, the 3D structures of carboxylic ester hydrolase, carboxylic ester hydrolase 2, GABA-RDL, GABA-GRD, GABA-gated ion channel LCCH3, glutamate-gated chloride channel, histamine-gated chloride channel 1, histamine-gated chloride channel 2, hormone receptor in 39-like, nicotinic acetylcholine receptor subunit alpha1, nicotinic acetylcholine receptor subunit alpha2, pH sensitive chloride channel, and putative octopamine/tyramine receptor proteins from *T. castaneum* were subsequently optimized and minimized using atomic partial charges with the Kollman method using the SYBYL 8.1.1 package (Tripos, San Luis, MO, USA). MGLTools 1.5.0 software (Molecular Graphics Laboratory, La Jolla, CA, USA) was utilized to convert structures from PDB to PDBQT format, adding polar hydrogens and assigning Kollman partial charges. The 3D structure of the linalool and β-pinene were obtained from Pubchem [55] and optimized using Gaussian 03 by quantum chemical calculations based in density functional theory (DFT) at the 6-311G level [56]. Docking studies were carried out using AutoDock Vina [57]. Docking was performed by establishing a cube of sufficient size to cover the complete protein with a grid point spacing of 1 Å. The average binding affinity for the best poses was accepted as the binding affinity value for a particular complex [56]. The existing ligand–residue interactions on the protein were evaluated with LigandScout 3.0 (Inte:Ligand, Maria Enzersdorf, Austria, Europe) [58]. The validation of the docking process was carried out by docking GABA, octopamine/tyramine and nicotinic receptors, and their inhibitors are reported in the literature (Table 2) [9,10].

### 4.7. Statistical Analysis

The paired *t*-test was utilized to compare the mean number of insects on the treated and untreated areas of the filter paper. Repellency or attraction was established if significant differences occurred for positive or negative percentage repellency, respectively. Probit analysis was employed to calculate RC_50_. Normal distribution and equality between variances were checked by the Kolmogorov–Smirnov and Bartlett’s tests, respectively. Two-way analysis of variance (ANOVA) with the Bonferroni post-test was used to compare all groups (treated and control) at different time intervals. The data are presented as means ± SE and the differences between means are considered to be significant at *p* ≤ 0.05. GraphPad InStat 3.05 (GraphPad Software, La Jolla, CA, USA) was used for data analysis. The parameters evaluated in the statistical analysis are found in Tables S1 and S2.

## 5. Conclusions

In summary, the monoterpenes linalool and β-pinene showed repellent activity against *T. castaneum*, affecting the expression of genes related to neurotransmission such as acetylcholinesterase, GABA-gated ion channel, and histamine-gated chloride channel 2. However, each chemical compound induced a different gene expression profile. These chemicals had little ability to dock on proteins associated with the neurotransmission process in the red flour beetle.

## Figures and Tables

**Table 1:** Results of the validation of homology-modeled proteins from *T. castaneum* using ProQ.

Proteins	LGscore	MaxSub
Carboxylic ester hydrolase	5.448	0.154
Carboxylic ester hydrolase 2 (acetylcholinesterase activity)	6.207	0.459
Gamma-aminobutyric acid-gated anion channel splice variant 3a6a (GABA-RDL)	1.520	0.087
Gamma-aminobutyric acid-gated ion channel (GABA-GRD)	2.658	0.120
Gamma-aminobutyric acid-ligand gated chloride channel 3) (GABA-LCCH3)	2.611	0.129
Glutamate-gated chloride channel	2.010	0.150
Histamine-gated chloride channel 1	2.487	0.166
Histamine-gated chloride channel 2	2.435	0.143
Hormone receptor in 39-like protein	0.947	0.432
Nicotinic acetylcholine receptor subunit alpha1	1.580	0.090
Nicotinic acetylcholine receptor subunit alpha2	1.707	0.118
pH sensitive chloride channel	1.740	0.139
Putative octopamine/tyramine receptor	2.150	0.100

**Table 2:** AutoDock vina-calculated affinities (Kcal/mol) obtained for docking linalool and β-pinene on some proteins.

Proteins	Uniprot Code	Linalool	β-Pinene	Inhibitors
Carboxylic ester hydrolase	E7DN61	−5.3 ± 0.1	−5.8 ± 0.0	
Carboxylic ester hydrolase 2 (acetylcholinesterase activity)	E7DN62	−5.5 ± 0.2	−6.1 ± 0.1	
Gamma-aminobutyric acid-gated anion channel splice variant 3a6a	A8DMU1	−4.9 ± 0.1	−6.1 ± 0.0	−9.8 ± 0.0 *
Gamma-aminobutyric acid-gated ion channel	A8DMU2	−5.1 ± 0.1	−6.0 ± 0.0	−9.4 ± 0.0 *
Gamma-aminobutyric acid-ligand gated chloride channel 3	A8DMU3	−6.7 ± 0.2	−7.2 ± 0.0	−9.0 ± 0.0 *
Glutamate-gated chloride channel	A8DMU5	−4.9 ± 0.2	−6.6 ± 0.3	
Histamine-gated chloride channel 1	A8DMU7	−5.3 ± 0.2	−5.6 ± 0.0	
Histamine-gated chloride channel 2	A8DMU8	−5.0 ± 0.2	−5.7 ± 0.0	
Hormone receptor in 39-like protein	D2A6H1	−5.2 ± 0.2	−6.0 ± 0.0	
Nicotinic acetylcholine receptor subunit alpha1	A8DIP3	−7.1 ± 0.2	−7.4 ± 0.1	−8.8 ± 0.0 ^+^
Nicotinic acetylcholine receptor subunit alpha2	A8DIQ7	−6.8 ± 0.3	−7.6 ± 0.0	−8.5 ± 0.0 ^+^
pH sensitive chloride channel	A8DMU9	−5.4 ± 0.2	−5.0 ± 0.0	
Putative octopamine/tyramine receptor	D6WB14	−7.2 ± 0.2	−7.7±0.0	−8.2 ± 0.0 ^‡^

* Ivermectin [9]; ^+^ Imidacloprid [10]; ^‡^ Promethazine [11].

**Table 3:** Primer sequences of the genes used for real-rime polymerase chain reaction (RT-PCR).

Gene Name	Gene Symbol	Entrez Gene ID	Forward (5′-3′)	Reverse (5′-3′)	Amplicon Size
**Genes Evaluated**					
Acetylcholinesterase	Ace1	HQ260968.1	CCGTTCGTCCCAGTCATTG	AGTAGTAGCCTTCTTCTGTGTTAG	121
GABA-gated anion channel splice variant 3a6a	Rdl	NM_001114292.1	ACTTGGGCGACGTCAACATA	ACGTGAAATCCATCTGGACC	159
GABA-gated ion channel	Grd	NM_001114300.1	GGTCTCCTTCTGGCTGAACC	TGGACCACAGCGAACTGAAT	198
Glutamate-gated chloride channel	Glucl	NM_001114304.1	TGAATGGCACAGATGGTCCC	CCAGACTCGACTGGCTTCAG	194
Histamine-gated chloride channel 2	Hiscl2	NM_001109951.1	TGGATGTCCAGTTGTTCGGT	TGTGGCTGAATAGGCAAGTCAT	176
Hormone receptor in 39-like protein	Hr39	XR_043083.1	CGACCGTCGACTGTACAAAA	AGTCGACATGGAACGGAAAC	145
**Housekeeping Gene**					
Ribosomal protein 49	Rps49	XM_964471.2	TGGCAAACTCAAACGCAACT	AGCGCCTACGAACCCTGTT	62
Ribosomal protein 18	Rps18	XM_968539.2	CGAAGAGGTCGAGAAAATCG	CGTGGTCTTGGTGTGTTGAC	235

## Supplementary Materials

Supplementary Materials are available online.
