# Supplementary material for: Neurotoxic Effects of Linalool and β-Pinene on Tribolium castaneum Herbst
Source: Molecules. 2017 Nov 24;22(12):2052. doi: 10.3390/molecules22122052 (PMC6149882; doi:10.3390/molecules22122052)
Supplement: Supplementary file 1 [file molecules-22-02052-s001.pdf]

Neurotoxic Effects of Linalool and β-Pinene on *Tribolium castaneum* Herbst

Nerlis Pajaro-Castro [1,2], Karina Caballero-Gallardo [1] and Jesus Olivero-Verbel [1,*]

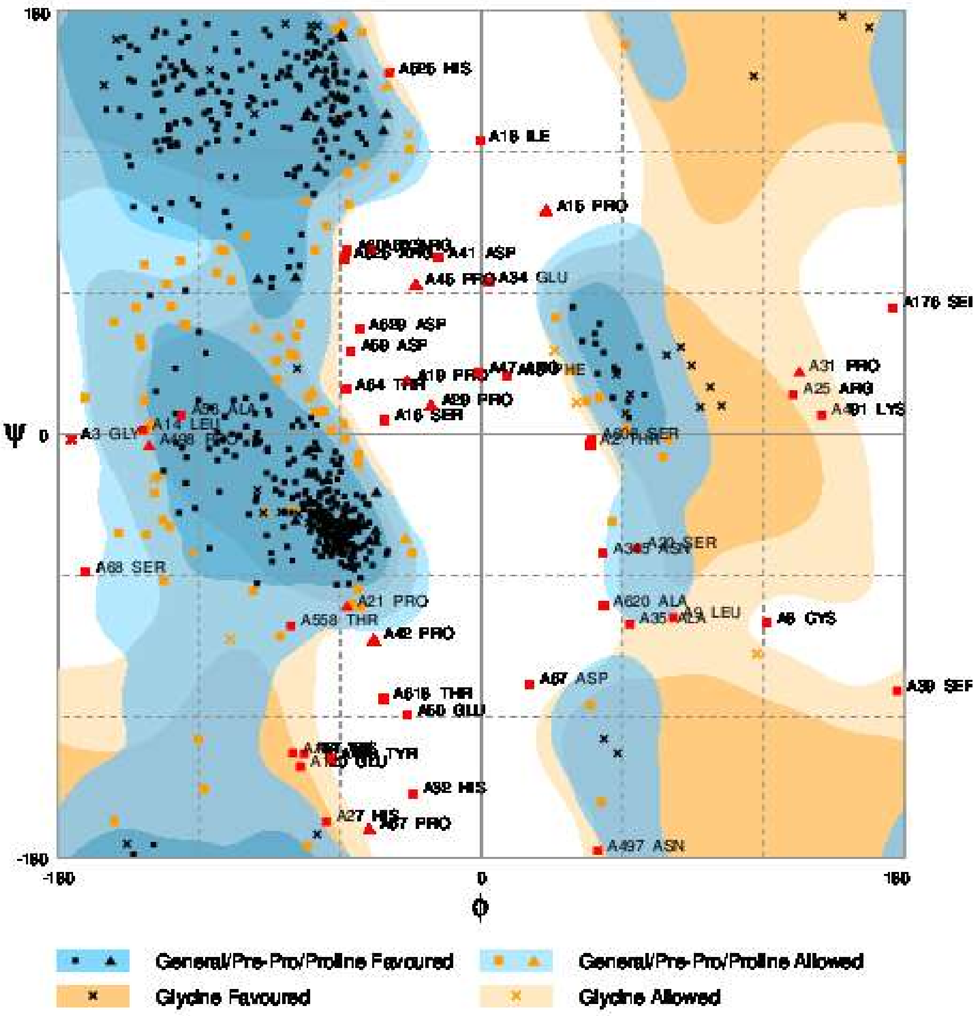

Figure S1. Ramachandran plot of the carboxylic ester hydrolase protein.

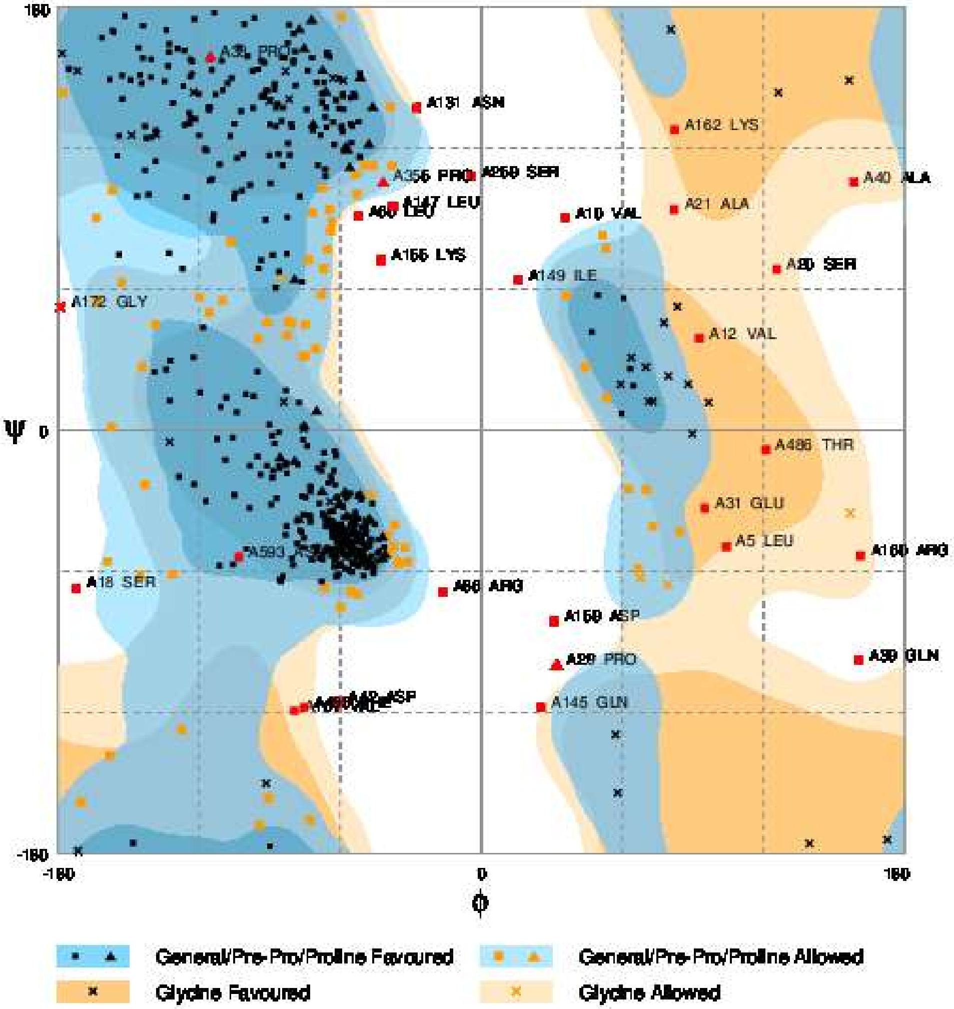

Figure S2. Ramachandran plot of the carboxylic ester hydrolase 2 protein.

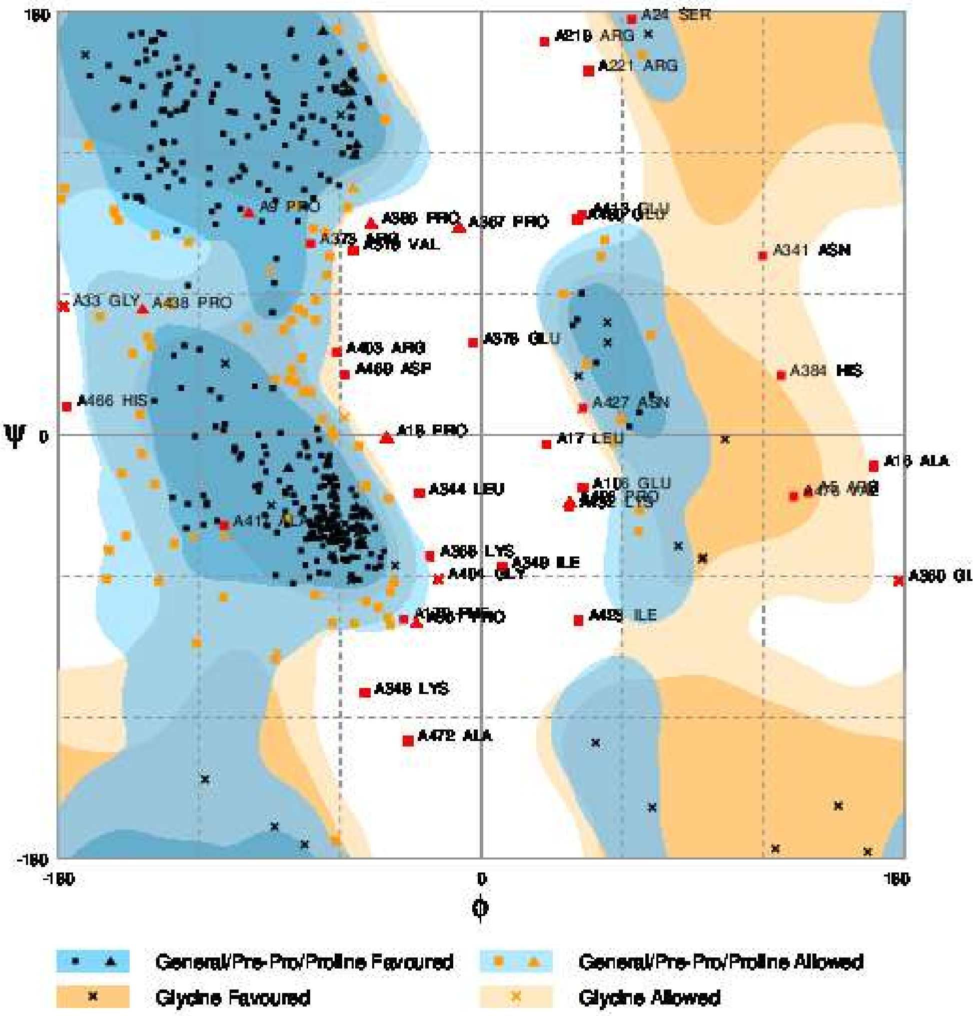

Figure S3. Ramachandran plot of the gamma-aminobutyric acid-gated anion channel splice variant 3a6a (GABA-RDL) protein.

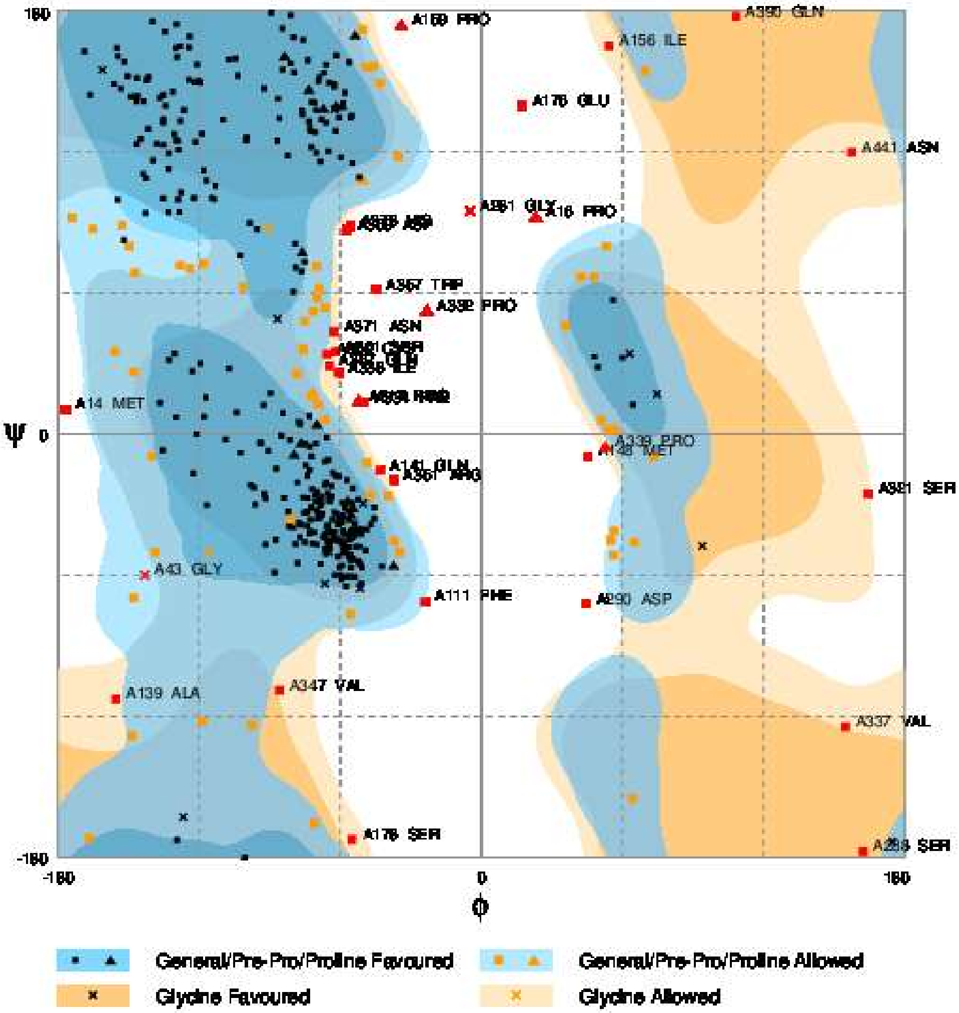

Figure S4. Ramachandran plot of the gamma-aminobutyric acid-gated ion channel (GABA-GRD) protein.

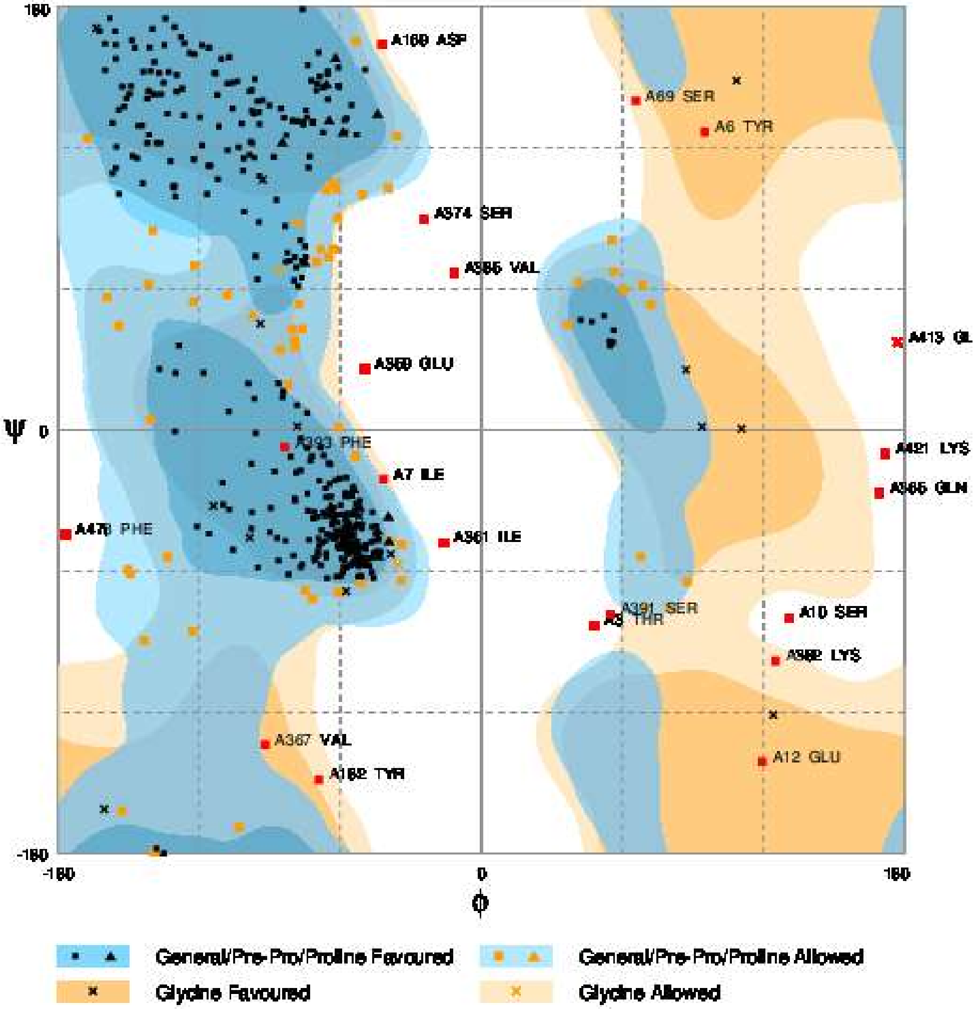

Figure S5. Ramachandran plot of the GABA-gated ion channel LCCH3 protein.

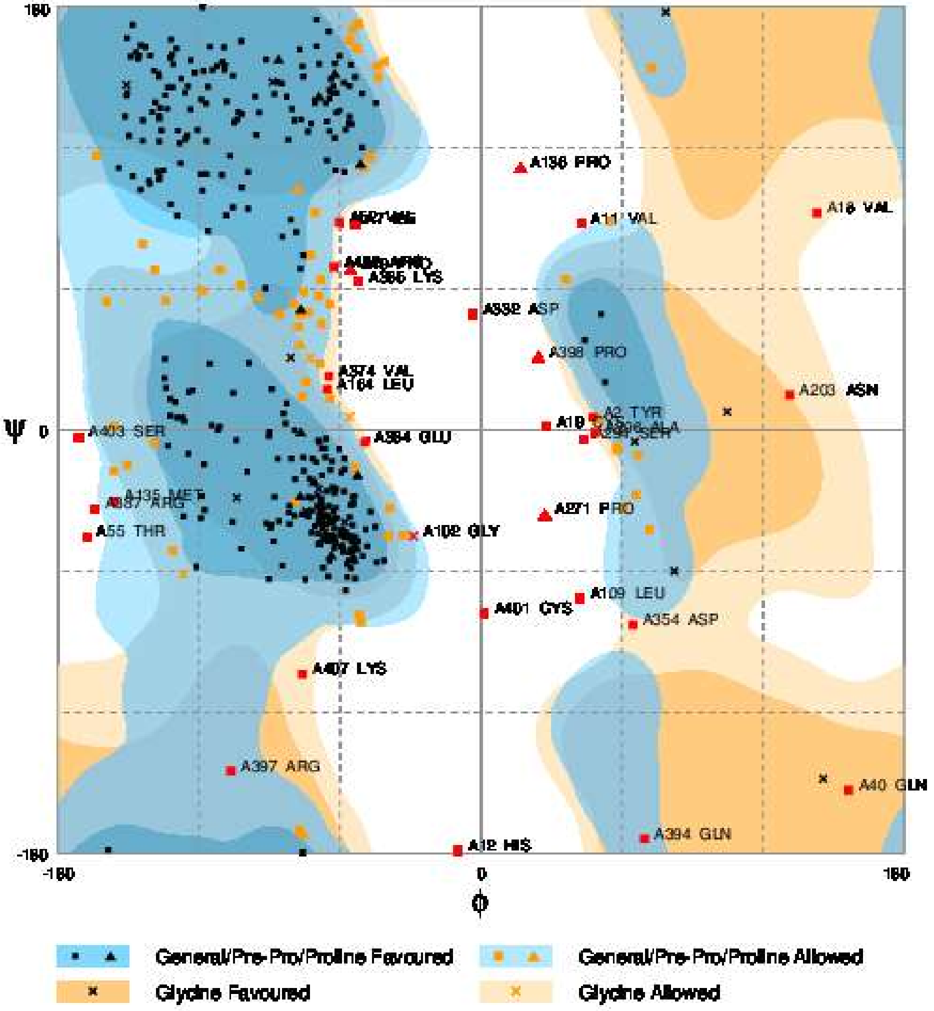

Figure S6. Ramachandran plot of the glutamate-gated chloride channel protein.

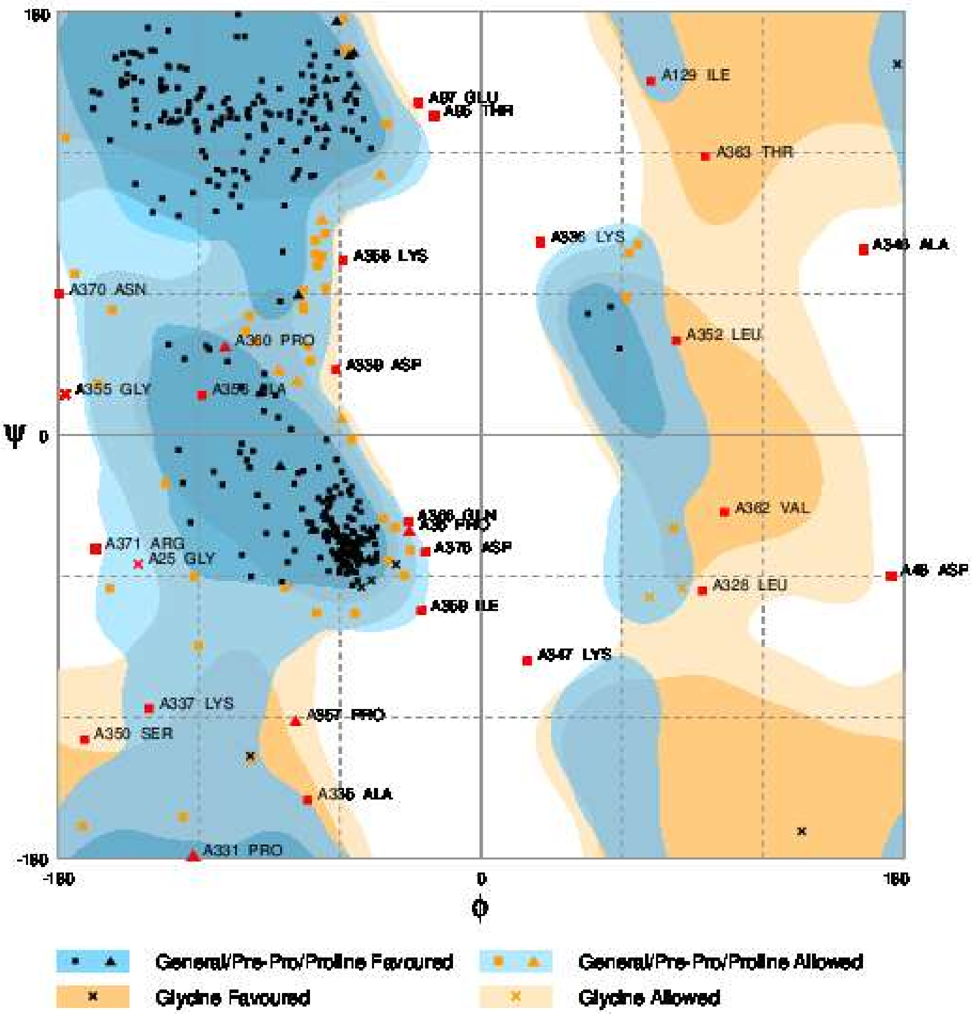

Figure S7. Ramachandran plot of the histamine-gated chloride channel 1 protein.

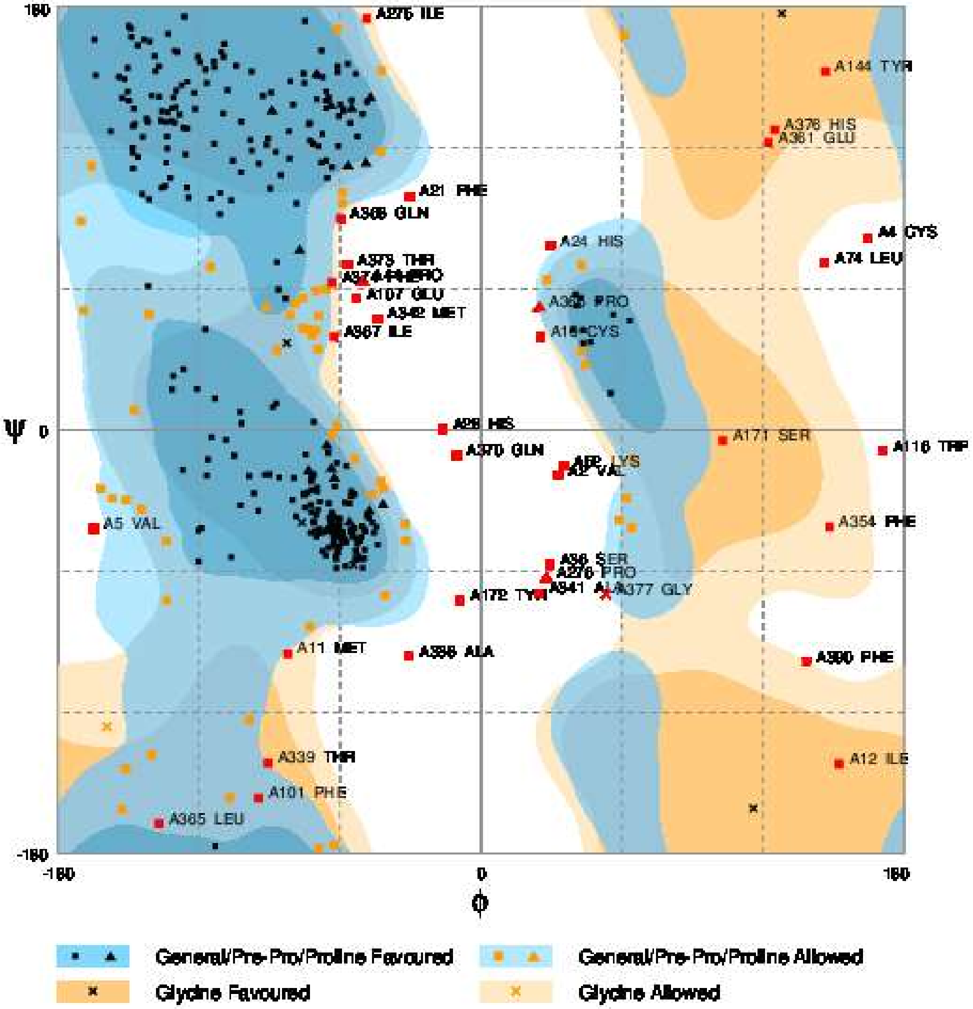

Figure S8. Ramachandran plot of the histamine-gated chloride channel 2 protein.

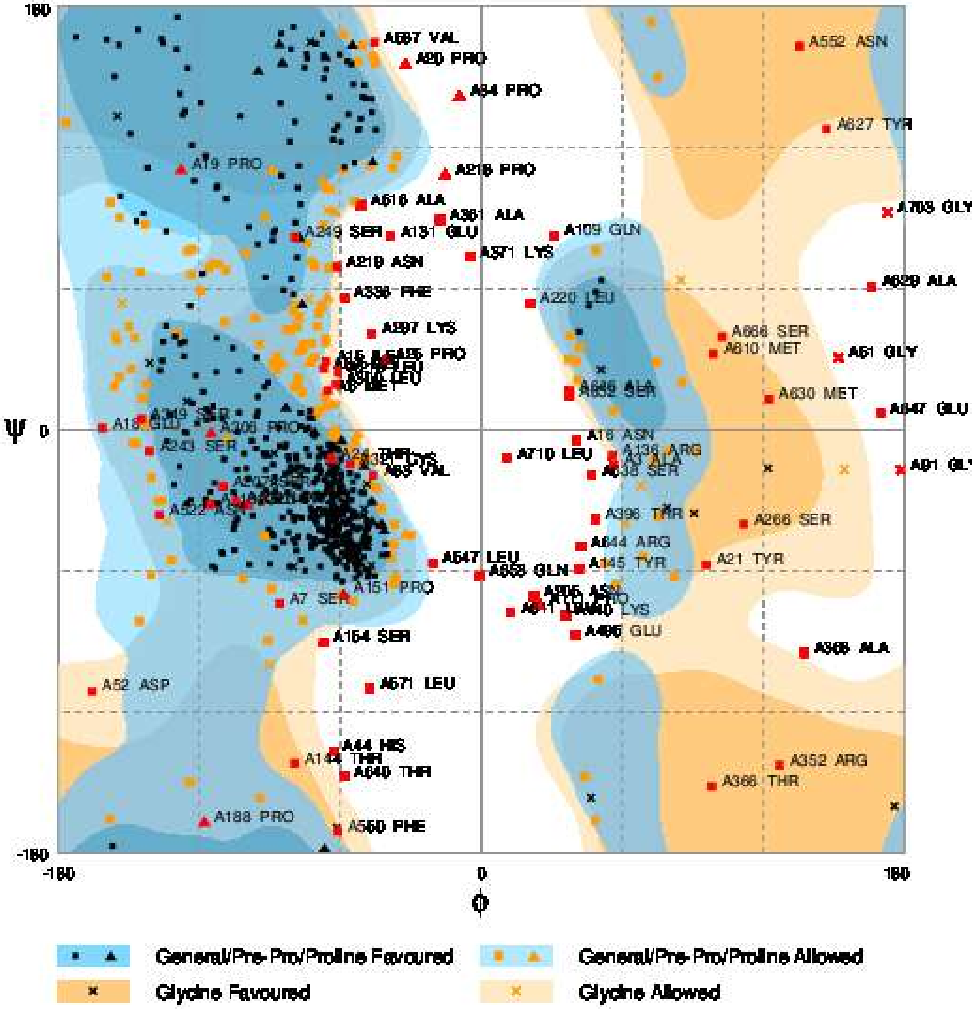

Figure S9. Ramachandran plot of the hormone receptor in the 39-like protein.

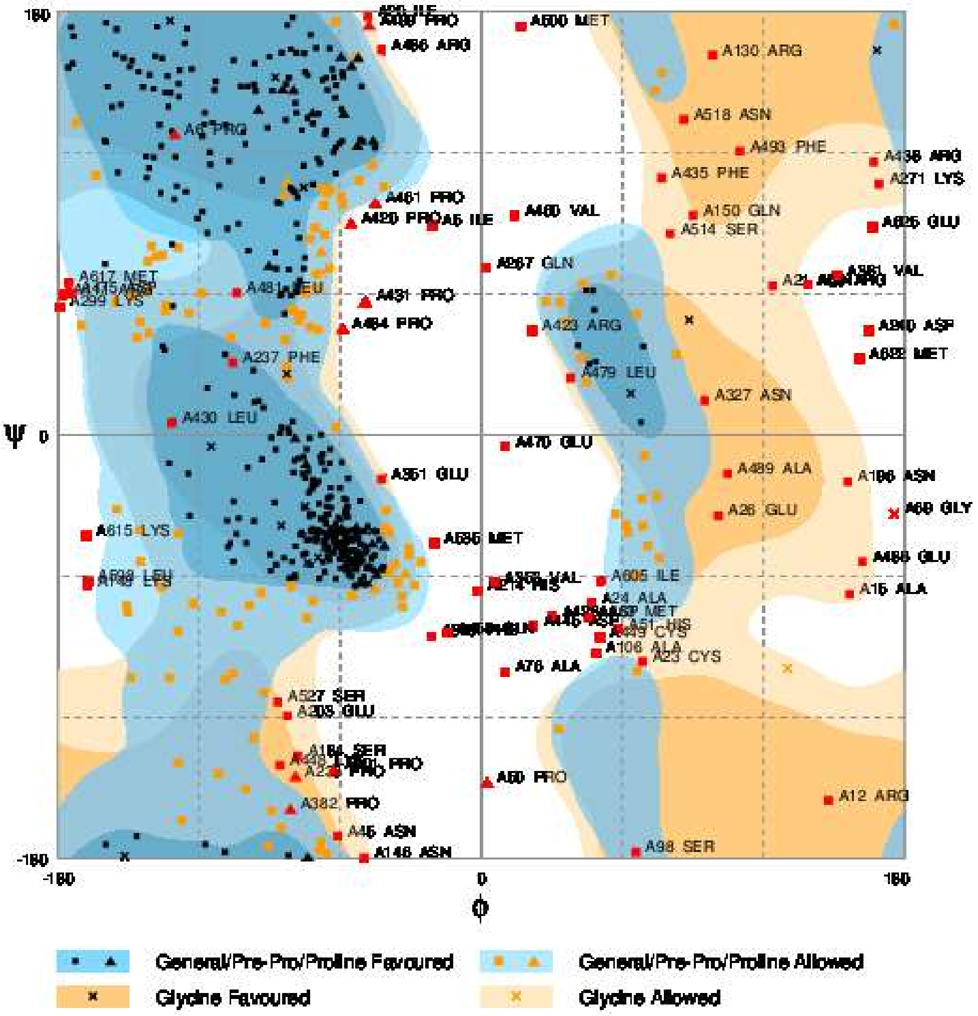

Figure S10. Ramachandran plot of the nicotinic acetylcholine receptor subunit alpha1 protein.

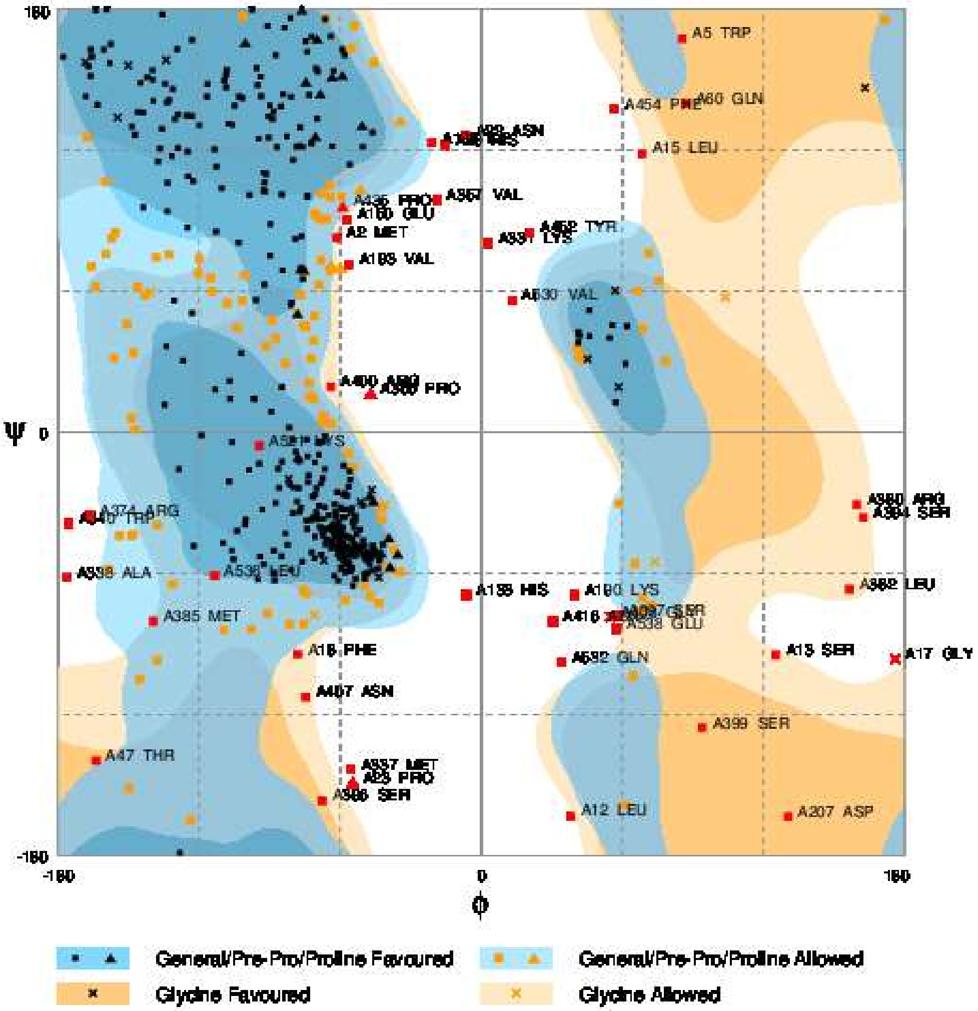

Figure S11. Ramachandran plot of the nicotinic acetylcholine receptor subunit alpha2 protein.

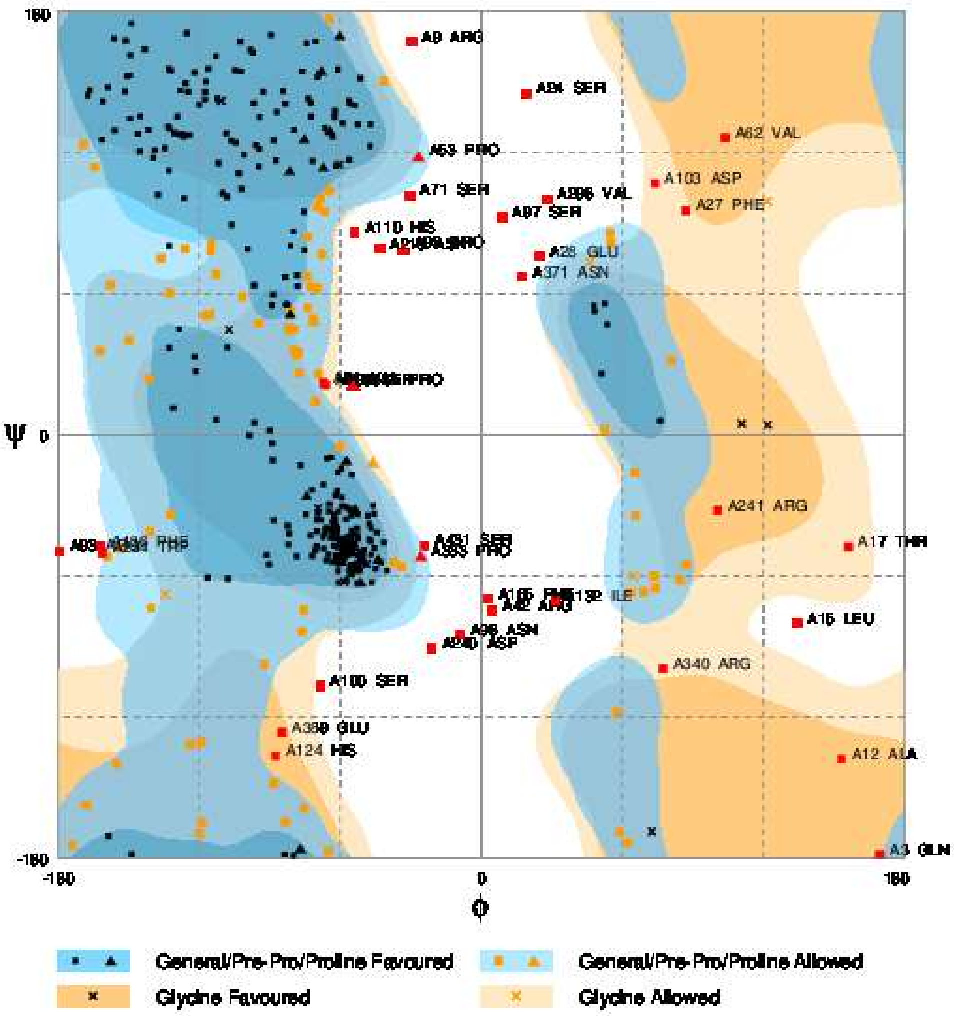

Figure S12. Ramachandran plot of the pH sensitive chloride channel protein.

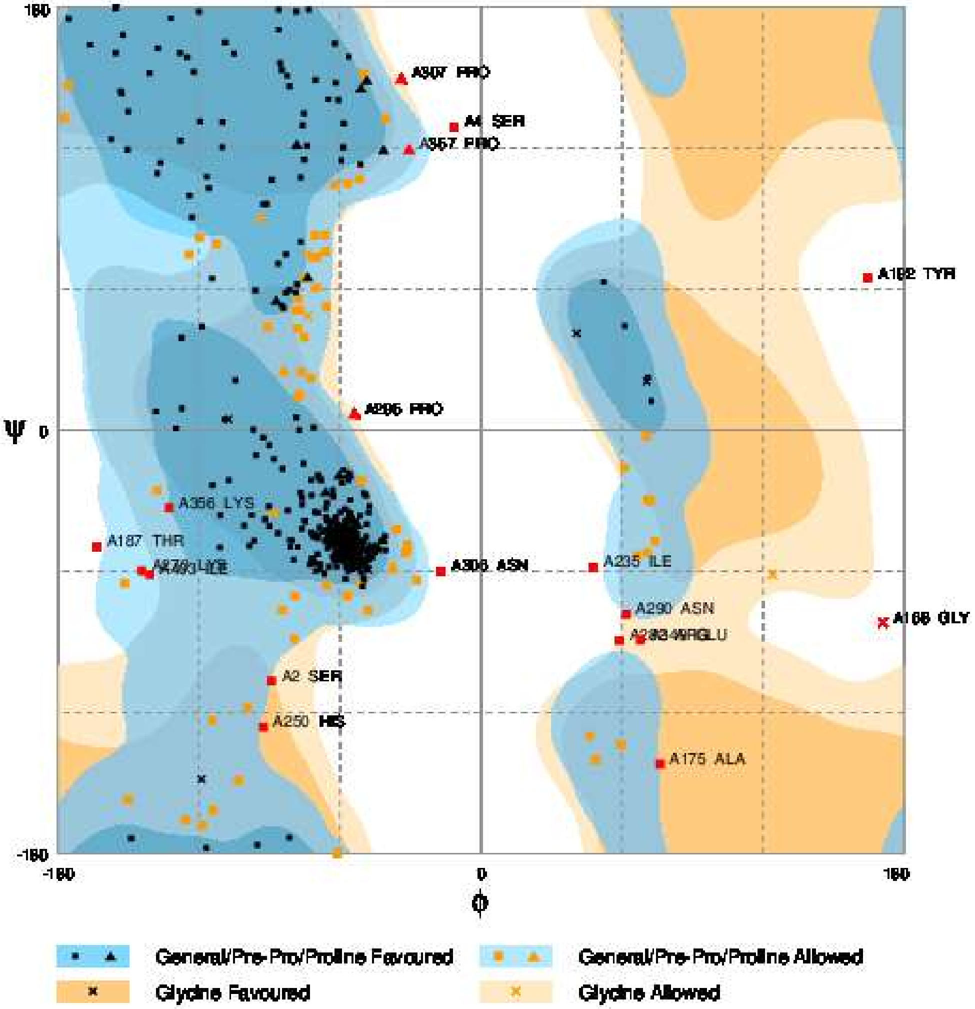

Figure S13. Ramachandran plot of the putative octopamine/tyramine receptor.

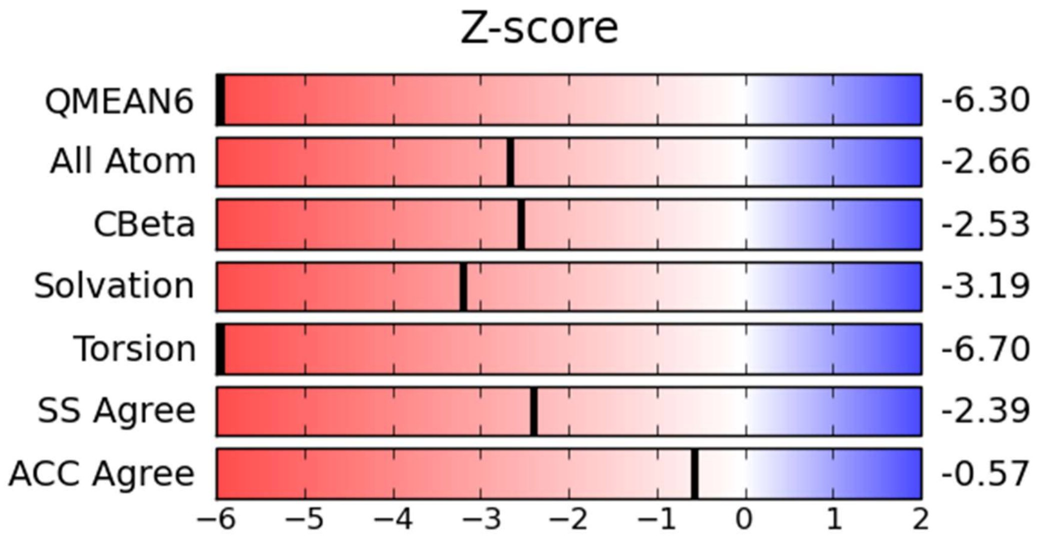

Figure S14. Z-score obtained by QMean for the carboxylic ester hydrolase protein.

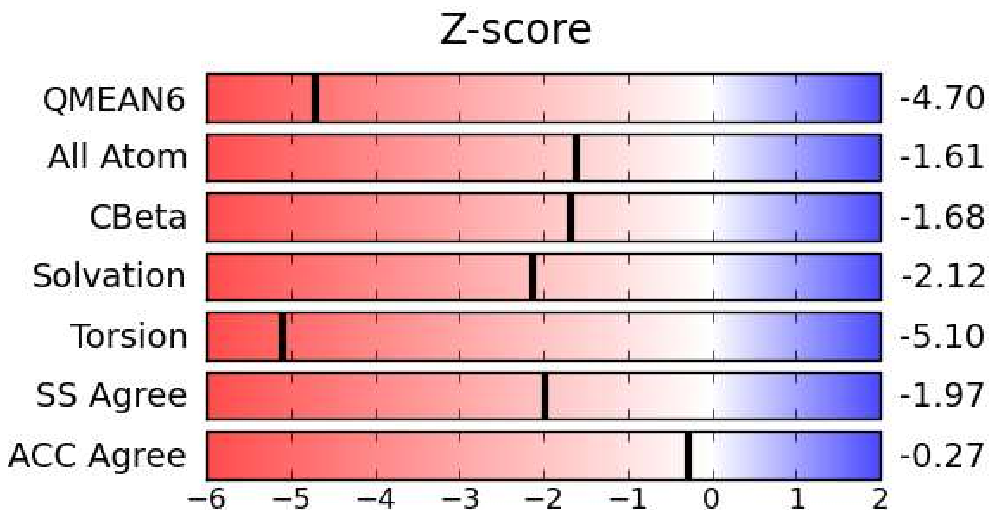

Figure S15. Z-score obtained by QMean for the carboxylic ester hydrolase 2 protein.

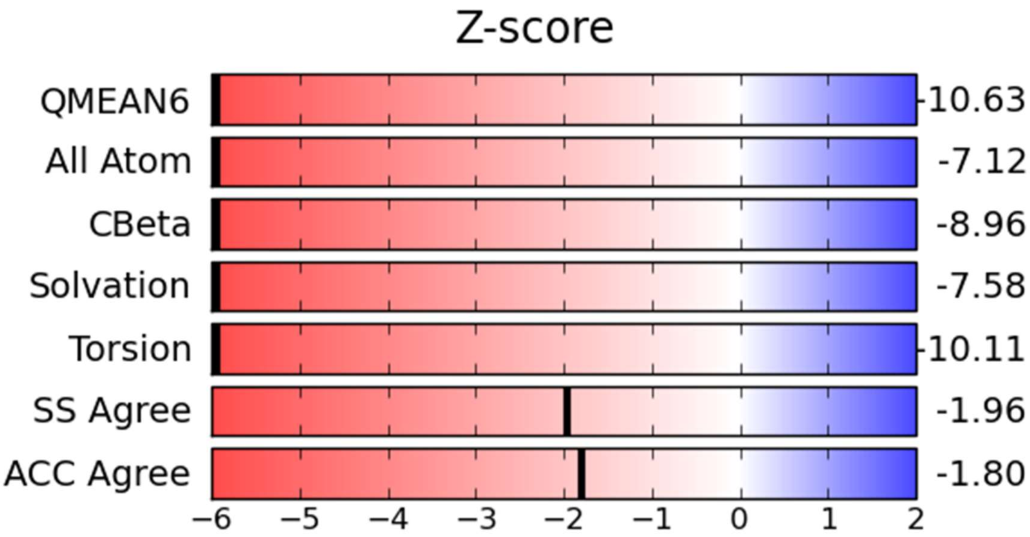

Figure S16. Z-score obtained by QMean for the gamma-aminobutyric acid-gated anion channel splice variant 3a6a (GABA-RDL) protein.

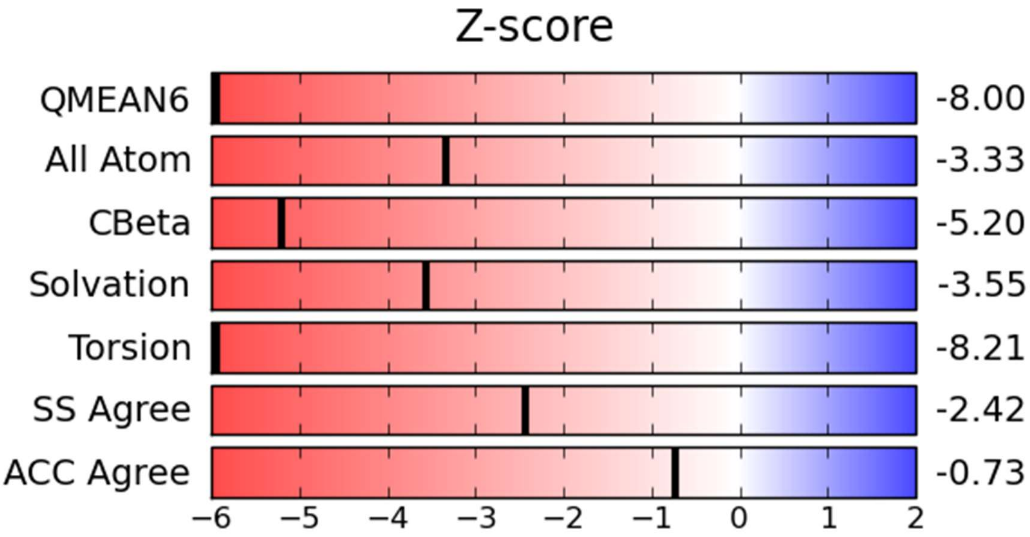

Figure S17. Z-score obtained by QMean for the gamma-aminobutyric acid-gated ion channel (GABA-GRD) protein.

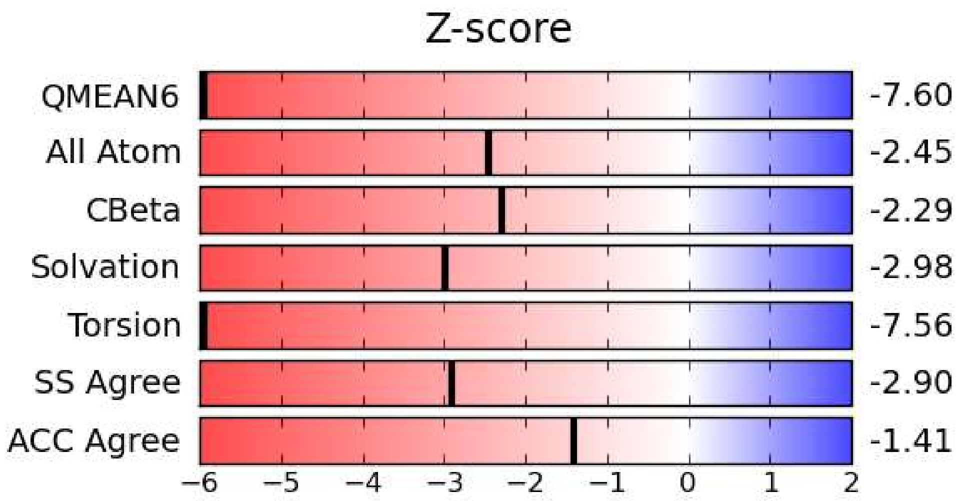

Figure S18. Z-score obtained by QMean for the GABA-gated ion channel LCCH3 protein.

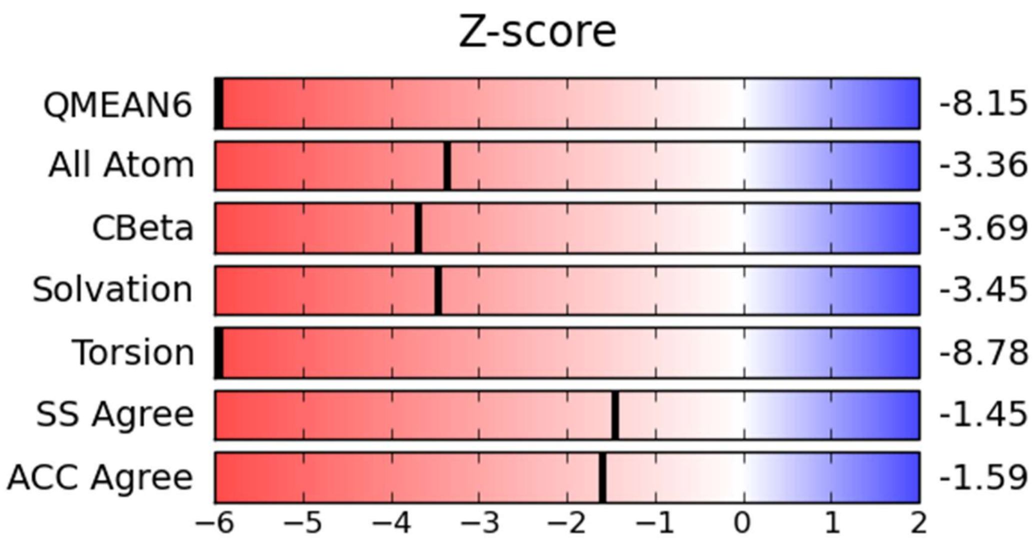

Figure S19. Z-score obtained by QMean for the glutamate-gated chloride channel protein.

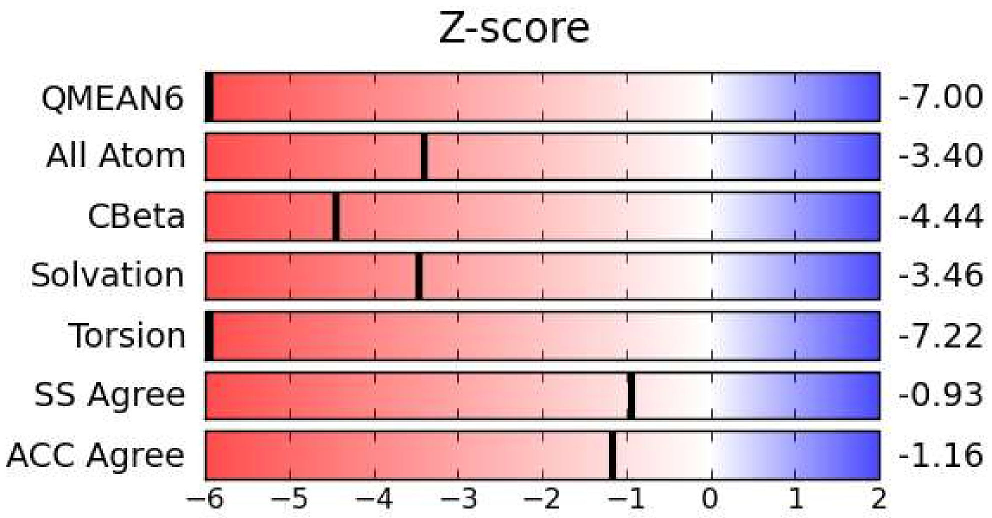

Figure S20. Z-score obtained by QMean for the histamine-gated chloride channel 1 protein.

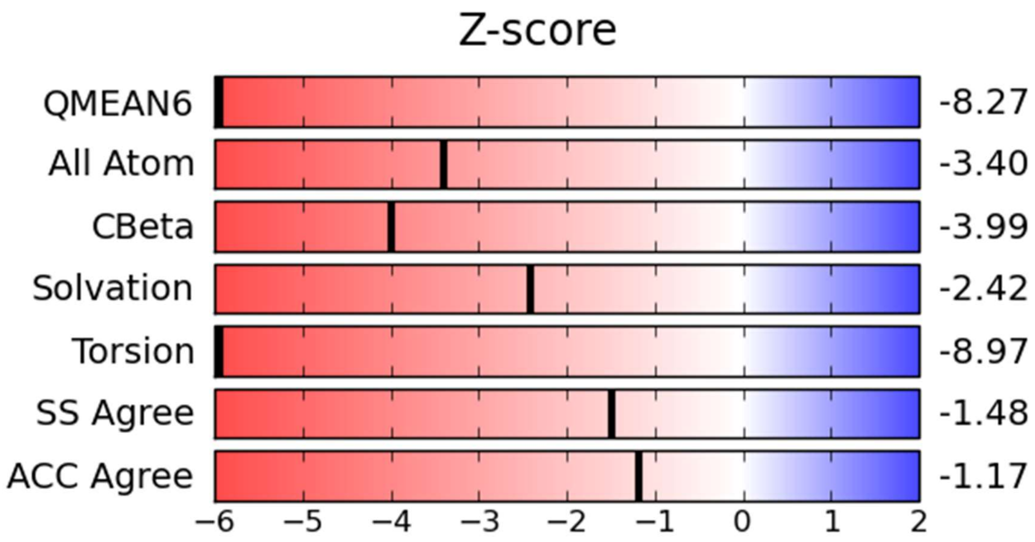

Figure S21. Z-score obtained by QMean for the histamine-gated chloride channel 2 protein.

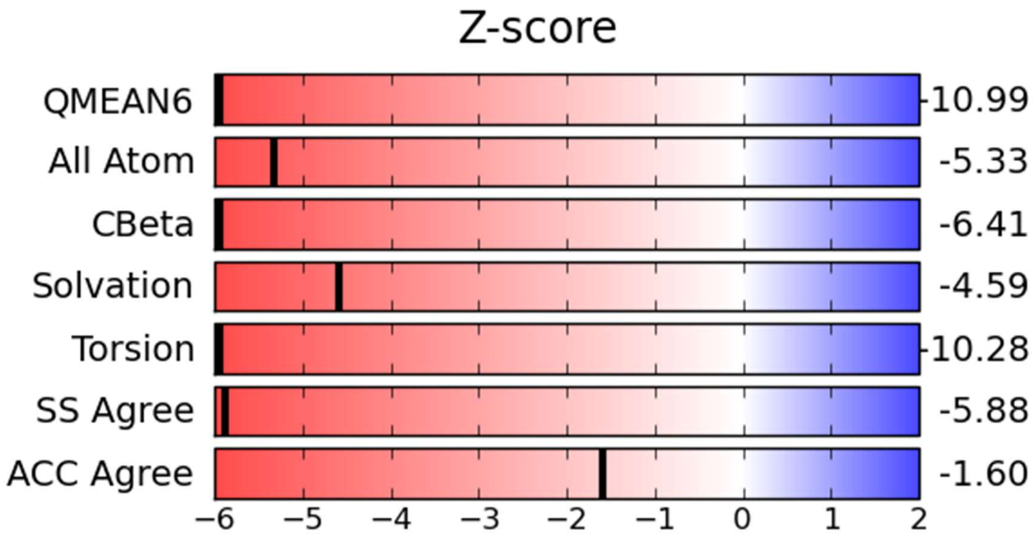

Figure S22. Z-score obtained by QMean for the hormone receptor in the 39-like protein.

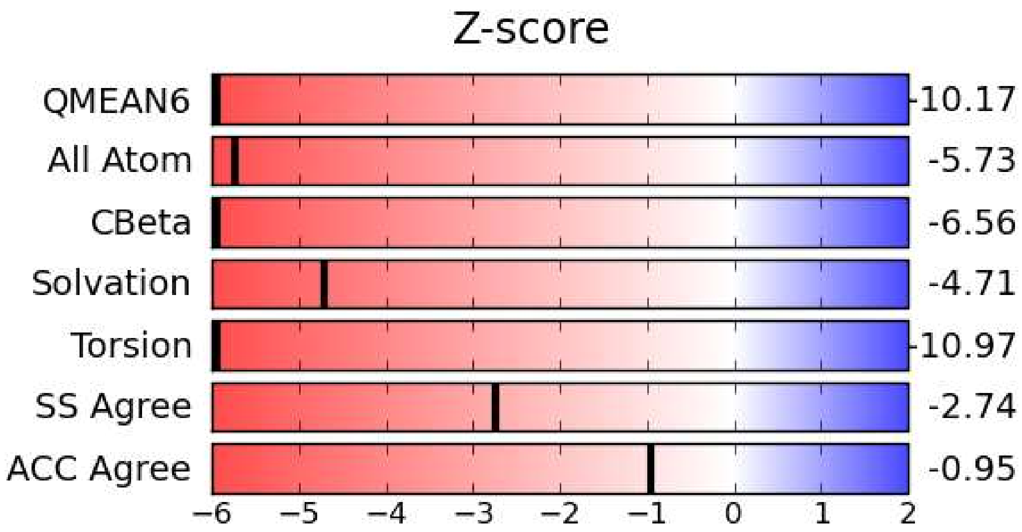

Figure S23. Z-score obtained by QMean for the nicotinic acetylcholine receptor subunit alpha1 protein.

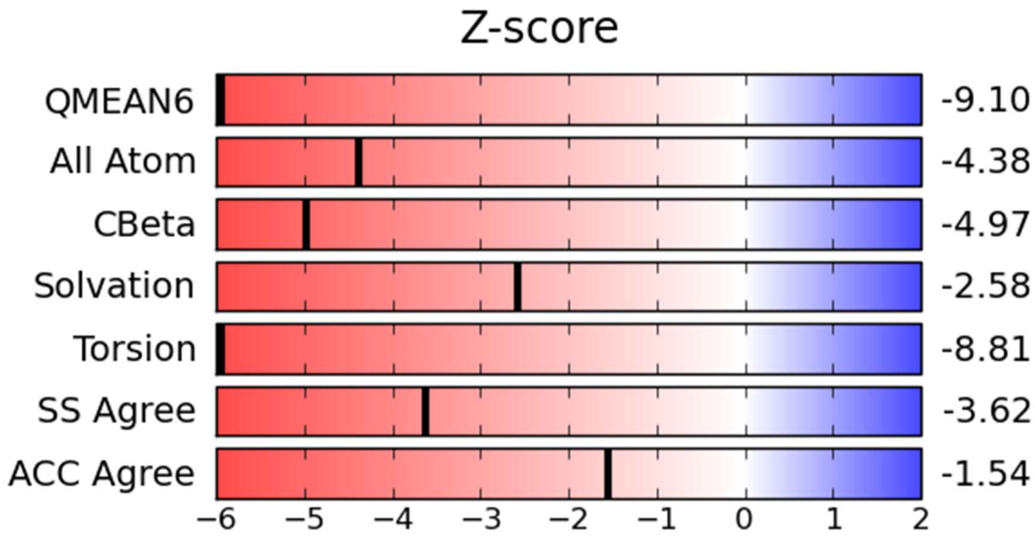

Figure S24. Z-score obtained by QMean for the nicotinic acetylcholine receptor subunit alpha2 protein.

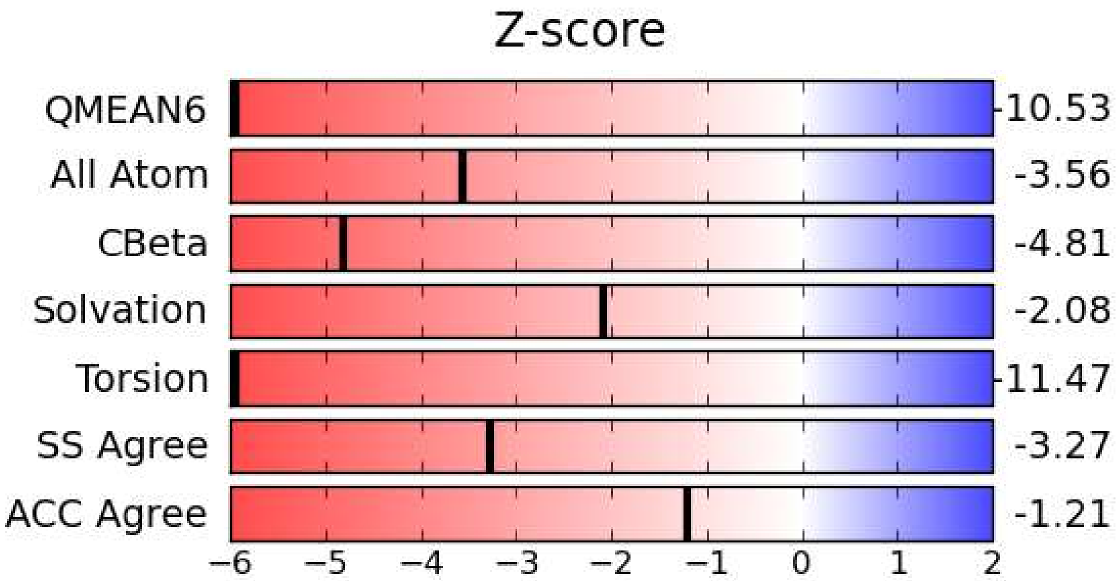

Figure S25. Z-score obtained by QMean for the pH sensitive chloride channel protein.

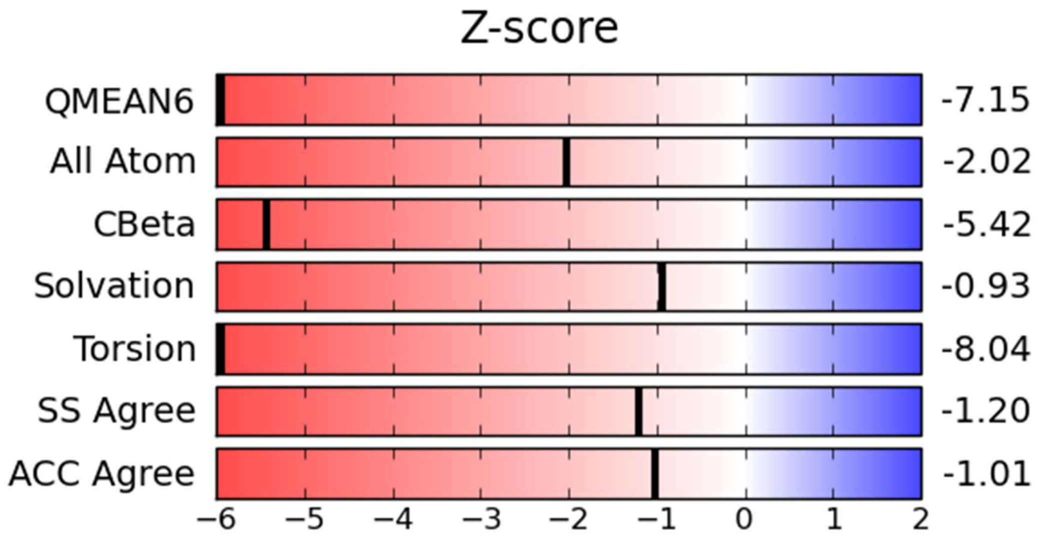

Figure S26. Z-score obtained by QMean for the putative octopamine/tyramine receptor.

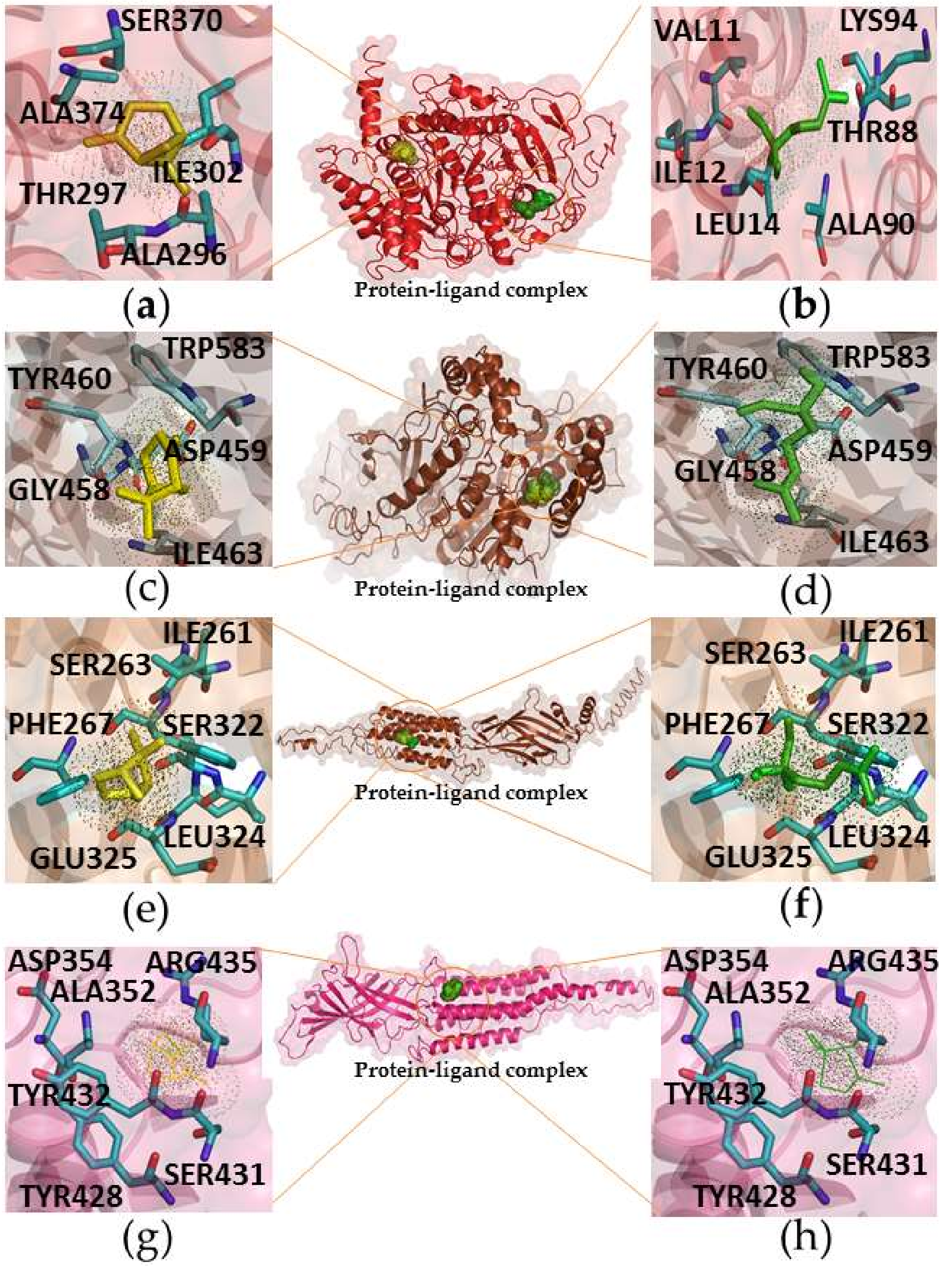
SER370
ALA374
ILE302
THR297
ALA296
(a)
Protein-ligand complex
VAL11
LYS94
THR88
ILE12
LEU14
ALA90
(b)
TRP583
TYR460
ASP459
GLY458
ILE463
(c)
Protein-ligand complex
TYR460
TRP583
ASP459
GLY458
ILE463
(d)
ILE261
SER263
PHE267
SER322
LEU324
GLU325
(e)
Protein-ligand complex
ILE261
SER263
PHE267
SER322
GLU325
LEU324
(f)
ASP354
ARG435
ALA352
TYR432
SER431
TYR428
(g)
Protein-ligand complex
ASP354
ARG435
ALA352
TYR432
SER431
TYR428
(h)

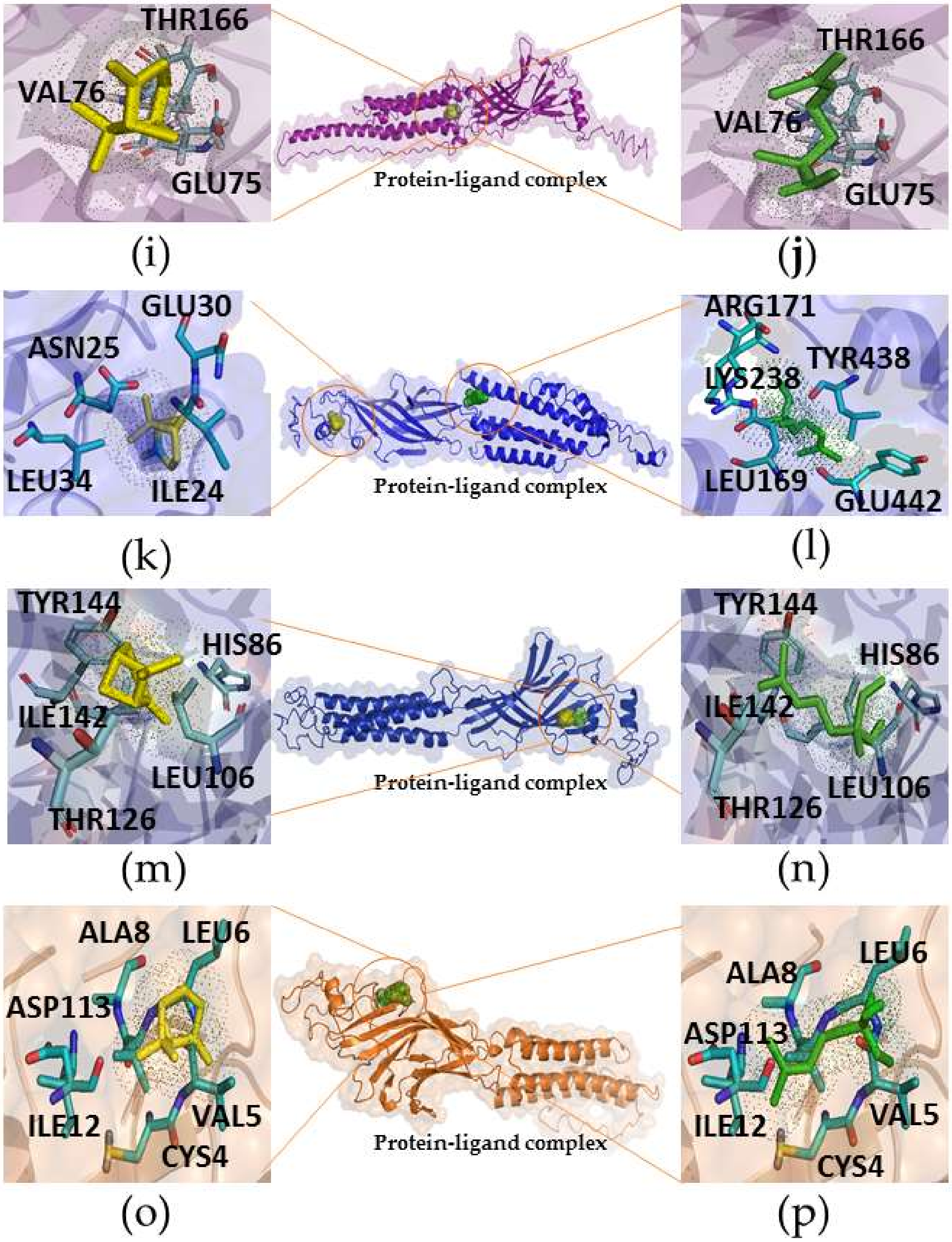
THR166
VAL76
GLU75
Protein-ligand complex
THR166
VAL76
GLU75
(i)
(j)
GLU30
ASN25
LEU34
ILE24
Protein-ligand complex
ARG171
TYR438
LYS238
LEU169
GLU442
(k)
(l)
TYR144
HIS86
ILE142
LEU106
THR126
Protein-ligand complex
TYR144
HIS86
ILE142
LEU106
THR126
(m)
(n)
ALA8
LEU6
ASP113
ILE12
VAL5
CYS4
Protein-ligand complex
LEU6
ALA8
ASP113
VAL5
ILE12
CYS4
(o)
(p)

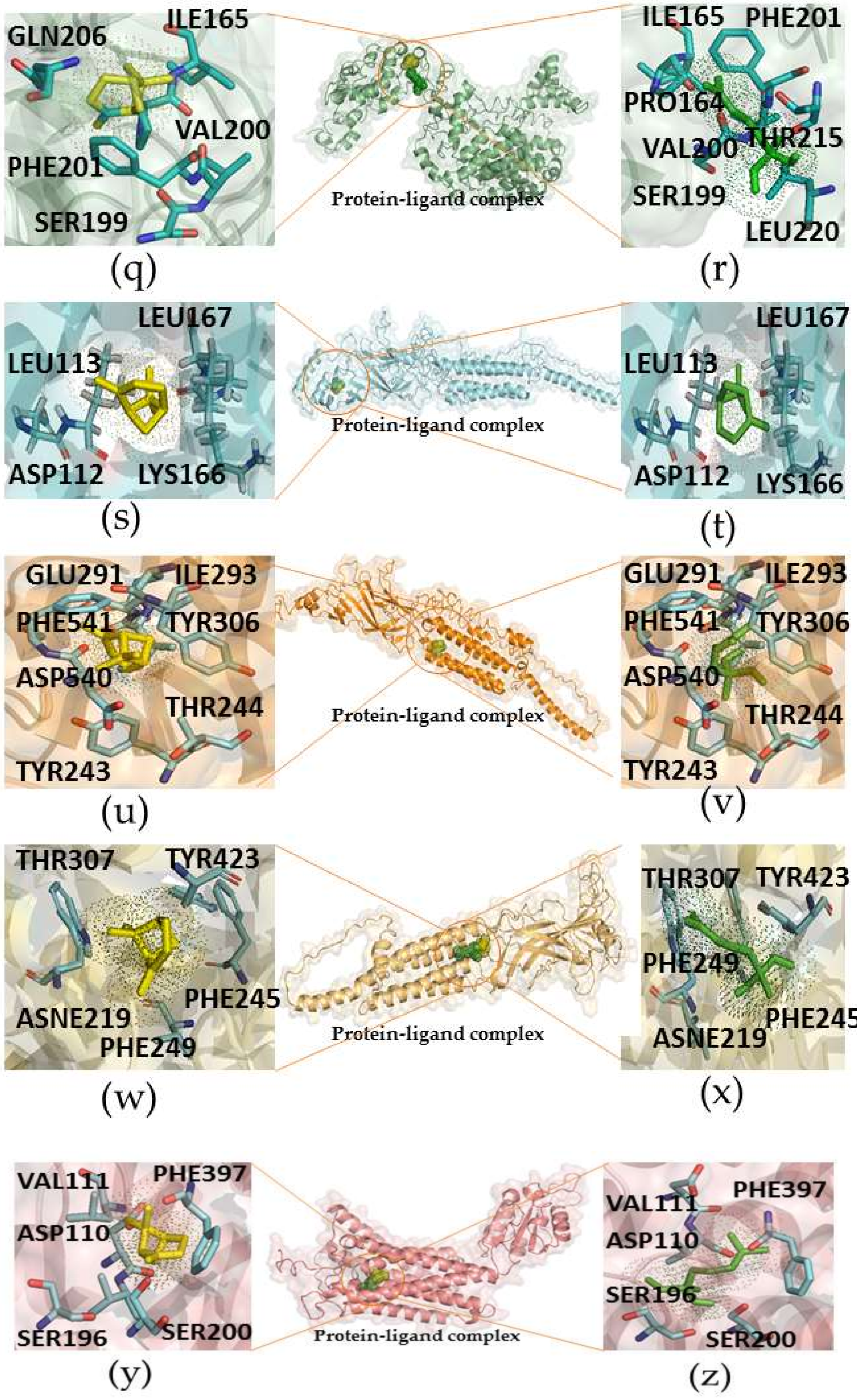

Figure S27. Predicted binding and interacting residues present in proteins from *T. castaneum*.

(**a**) carboxylic ester hydrolase-β-pinene (yellow) complex; (**b**) carboxylic ester hydrolase-linalool (Green) complex; (**c**) carboxylic ester hydrolase2-β-pinene complex; (**d**) carboxylic ester hydrolase2-linalool complex; (**e**) GABA-RDL-β-pinene complex; (**f**) GABA-RDL-linalool complex; (**g**) GABA-GRD-β-pinene complex; (**h**) GABA-GRD- linalool complex; (**i**) GABA-gated ion channel LCCH3-β-pinene complex; (**j**) GABA-gated ion channel LCCH3- linalool complex; (**k**) glutamate-gated chloride channel-β-pinene complex; (**l**) glutamate-gated chloride channel-linalool complex; (**m**) histamine-gated chloride channel 1-β-pinene complex; (**n**) histamine-gated chloride channel 1-linalool complex; (**o**) histamine-gated chloride channel 2-β-pinene complex; (**p**) histamine-gated chloride channel 2-linalool complex; (**q**) hormone receptor in the 39-like protein-β-pinene complex; (**r**) hormone receptor in the 39-like protein-linalool complex; (**s**) nicotinic acetylcholine receptor subunit alpha1-β-pinene complex; (**t**) nicotinic acetylcholine receptor subunit alpha1-linalool complex; (**u**) nicotinic acetylcholine receptor subunit alpha2-β-pinene complex; (**v**) nicotinic acetylcholine receptor subunit alpha2-linalool complex; (**w**) pH sensitive chloride channel-β-pinene complex; (**x**) pH sensitive chloride channel-linalool complex; (**y**) putative octopamine/tyramine receptor-β-pinene complex; and (**z**) putative octopamine/tyramine receptor-linalool complex.
